# Unburnable carbon in the rapidly warming Arctic: Mapping spatial relationships among oil and gas development, ecologically sensitive areas and Indigenous Peoples’ lands

**DOI:** 10.1371/journal.pone.0345775

**Published:** 2026-04-22

**Authors:** Daniele Codato, Daniele Vezzelli, Federica Ammaturo, Giorgia Lazazzera, Andrea Stralla, Salvatore E. Pappalardo, Massimo De Marchi

**Affiliations:** 1 Department of Civil, Environmental and Architectural Engineering, University of Padova, Padova, Italy; 2 Department of Geography, Humboldt-Universität zu Berlin, Berlin, Germany; 3 Leibniz Institute for Research on Society and Space (IRS), Erkner, Germany; 4 ENGIM NGO, Roma, Italy; 5 Independent Landscape Consultant, Cuneo, Italy; MARE – Marine and Environmental Sciences Centre, PORTUGAL

## Abstract

As global temperatures rise due to carbon emissions from fossil fuels, it is estimated that around 60% of oil and gas reserves —termed 'unburnable carbon'—must remain underground to meet international climate targets. The Arctic, warming nearly four times faster than the global average, is a frontline for both climate change impacts and oil and gas development, which pose severe risks to its fragile biodiversity and Indigenous communities. This study presents the first comprehensive spatial atlas of Arctic oil and gas infrastructure using open-access data, assessing overlaps with ecologically sensitive areas and Indigenous Peoples’ lands (IPLs) within the Conservation of Arctic Flora and Fauna (CAFF) boundary. The analysis identified 512,306 km^2^ of exploited territory (1.82% of the Arctic region), 44,539 wells, 39,535 km of pipelines, and over 1.95 million km of seismic lines. Results show that 73.30% of hydrocarbon areas intersect IPLs and 7.57% overlap protected areas, with developments located in close proximity to key ecologically sensitive areas and culturally significant sites. These findings highlight the spatial overlaps and potential tensions between oil and gas industry interests and those behind the geographies of ecologically sensitive areas and IPLs, especially in zones such as Alaska’s North Slope and Russia’s Yamal Peninsula. The study calls for a paradigm shift in Arctic governance, from resource extraction toward equity, ecological preservation, and Indigenous inclusion. It supports the implementation of Arctic-specific supply-side climate policies, such as establishing an Arctic Fossil Fuel Non-Proliferation zone, to prevent further ecological degradation and to ensure a just transition. By integrating spatial justice criteria into decision-making, this work provides a tool for guiding sustainable and inclusive land-use and energy transition planning across the region.

## 1 Introduction

Carbon dioxide emissions produced by fossil fuel extraction and combustion are widely recognized as the primary contributors to climate change and global warming, accounting for approximately 65% of the GHGs total emissions in 2019 [1]. In 2023, during the controversial COP 28 in Dubai, nations acknowledged for the first time the urgent need to transition away from fossil fuels in energy systems in a just, orderly and equitable manner to meet the goals outlined in the Paris Agreement, that set the target of limiting global warming to 1.5°C to mitigate the most severe impacts of climate change [2].

To keep global warming within a 1.5°C consistent pathway, urgent cuts on the supply of fossil fuels are required: around 60% of the oil, 60% of gas and 90% coal reserves must remain unextracted and unburned by 2050 [3]. This concept, known in literature as ‘unburnable carbon/fossil fuels’ or ‘unextractable fossil fuels’, defines the quantity of known reserves that must remain underground to avoid exceeding internationally agreed temperature limits. Building on this concept, different studies have underlined that any new development of oil and gas fields and coal mines is not compatible with the Paris threshold [4,5]. These findings become even more significant when considering that current estimates likely overestimate the remaining carbon budget for a fossil fuel phase-out [6]. Furthermore, from a legal and economic perspective, the 2025 International Court of Justice [7] climate advisory opinion and updated analyses of equitable, per-capita, and responsibility-based carbon budgets show that industrialized countries have already exceeded their fair-share emissions allocations [8–10]. This overuse implies a duty to avoid initiating new fossil fuel exploration and extraction, especially in highly sensitive regions.

In this framework, a growing number of supply-side initiatives advocate the establishment of an international agreement to stop the proliferation of fossil fuel production and to keep unburnable carbon underground [11]. One of the most widespread initiatives involving countries, municipalities, public institutions, and NGOs is represented by the Fossil Fuel Non-Proliferation Treaty initiative [12].

In this pathway towards the phase-out of fossil fuel production, the debate regarding spatial conflicts among the expansion of the oil and gas frontier and areas of conservation priority for biological and cultural diversity is becoming increasingly crucial [13–17], as recently proved by the historic referendum to halt oil and gas activities in the megadiverse Yasuní National Park in the Ecuadorian Amazon [18]. In this regard, previous studies have already investigated relationships between oil and gas activities and highly sensitive areas and tested methodologies and geographical criteria to identify the explicit location of the unburnable carbon, particularly in key globally recognized biocultural areas, such as the Amazon region [16,19].

Among these globally sensitive areas, the Arctic region has become central in the public discussions about the implementation of environmental conservation and climate policies aimed at keeping fossil fuels underground, as shown by the moratoria on offshore activities in Alaska and Canada and the stop to new exploration activities in Greenland [20,21]. The Arctic, which covers 5% of the Earth’s land surface, alongside the Sub-Arctic, hosts more than 50% of the world’s wetlands and is home to key biodiversity [22]. While there is not a unique definition of the Arctic, it occupies vast land, marine, and ice areas across Alaska (USA), Canada, Finland, Greenland (Denmark), Iceland, Norway, Russia, and Sweden.

In this region, the impacts of climate change are being observed with more severe consequences and earlier than the rest of the world. Warming in the Arctic is occurring at a rate four times faster than the global average [23]. The impacts of a warming atmosphere on the physical, chemical, biological and human components of Arctic ecosystems are numerous and well documented. These range from direct effects, such as melting of sea ice, sea level rise and permafrost thaw, to secondary effects such as coastal erosion and decreasing of surface reflectivity, as well as accelerated ocean warming [24,25]. The seasonal retreat of sea ice is expanding open water windows, facilitating increased industrial development, particularly oil and gas exploration and extraction [25]. This phenomenon, where climate change enables further fossil fuel exploitation that then exacerbates global warming, is often referred to as the 'Arctic paradox' [26,27].

The hydrocarbon rush in the Arctic has been also significantly driven by the estimates presented in the United States Geological Survey (USGS) Circum-Arctic Resource Appraisal and its accompanying 2008–3049 Factsheet [28,29]. These reports portrayed the Arctic as a resource-rich region, estimating it may hold approximately 30% of the world’s undiscovered natural gas and 13% of its undiscovered oil. This narrative quickly gained traction, with the USGS findings widely cited in media and policy documents, frequently without acknowledging the uncertainty of these estimations, fuelling international interest and geopolitical competition [30]. As a result, the USGS estimates have become deeply embedded in global policy discourse, including in the Arctic strategies of non-Arctic states. Science diplomacy, rather than guiding the debate toward climate governance, has instead reinforced a narrative that balances climate change concerns with the pursuit of resource exploitation [30].

Although the Arctic is identified as one of the regions where oil and gas resources should be left completely undeveloped [3], regional oil and gas production accounted for 5.5% of the global production in 2022 and is expected to further increase in the next few years [31].

The impacts of hydrocarbon extraction are well-documented and extend beyond carbon emissions and climate change [32,33]. They also include those related to the overall footprint of exploration and extraction activities infrastructure. This process can be summarized in four main phases: hydrocarbon prospecting, well perforation and pipeline construction, well exploitation, decommissioning. At each stage, a wide variety of effects on the environment can be observed, as much in terms of modification of the ecosystems as in contamination of soil, waters and air [32–34]. Importantly, even before extraction begins, the construction of roads and other infrastructure already causes significant disruption to Arctic biodiversity. Seismic explorations provoke sounds and vibrations that confound whales [35] whereas onshore seismic prospecting can cause habitat disturbance, soil compaction, and long-lasting impacts on terrestrial ecosystems and local communities [36,37]. Moreover, wells and pipelines development is changing reindeers’ migratory routes [38,39] while transportation ships traffic is increasingly affecting narwhals, walruses and many other marine species [40,41]. Ultimately, Arctic marine ecosystems are particularly vulnerable to oil spills due to their slow recovery rates [42,43].

Beyond ecological concerns, oil and gas development also plays a role in the lives of several Arctic communities, particularly Indigenous Peoples, who represent approximately 10% of the 4–10 million Arctic residents [44]. International concern over the lands and rights of native populations is reflected in international legal frameworks such as ILO Convention No. 169 [45] that obliges states to safeguard Indigenous Peoples rights to their lands, territories, and natural resources, and to guarantee meaningful participation, through free, prior, and informed consent, in decisions related to resource extraction. In fact, beyond the immediate risks of pollution and food insecurity [25], hydrocarbon activities pose existential threats to cultural practices of several Arctic Indigenous communities. These include the loss of sacred lands, disruption of semi-nomadic lifestyles such as reindeer herding, and erosion of cultural identity [46,47]. Moreover, although some Arctic regions experience short-term economic benefits from hydrocarbon projects, such as employment opportunities and infrastructure development, Cassotta and Mazza [48] emphasize that these gains are often concentrated in external or state-level actors, while Indigenous and local populations remain marginalized, both in terms of financial returns and participation in decision-making. This disparity highlights a structural imbalance in Arctic energy governance, where social impact assessments frequently fail to incorporate Indigenous perspectives meaningfully and where existing legal frameworks offer limited mechanisms for contesting or shaping development outcomes. As a result, the distribution of environmental risks and economic benefits remains highly uneven, raising serious concerns about distributive and procedural justice, recognition, cultural survival, and the legitimacy of governance processes in the region [44,48].

While opposition to oil and gas projects by Indigenous communities is well documented, spatial overlaps or proximity between extractive activities and Indigenous territories do not always translate into conflictual issues, as shown by those communities that have engaged in negotiated and benefit-sharing arrangements with industry, as observed, for example, in parts of Alaska’s North Slope region and Arctic Canada [44,49]. At the same time, these cases do not imply unanimous or unconditional support for oil and gas development within Indigenous communities. Rather, they reflect context-specific decisions shaped by historical, economic, and political conditions. By contrast, there is a strong normative and legal basis for prioritizing bans on oil and gas activities and identifying ‘unburnable carbon’ areas where extraction overlaps with the territories of Indigenous Peoples who have not provided free, prior, and informed consent, thereby constituting a violation of Indigenous land rights and self-determination [50].

This study adopts a geographical approach to investigate the spatial relationships and interactions among fossil fuels activities, ecologically sensitive areas and Indigenous Peoples’ lands (IPLs) across the entire Arctic Region, in order to support decision-making processes for energy transition and unburnable carbon policies. To achieve this, a methodology based on Geographical Information Systems (GIS) is used. Specific objectives for the whole Arctic region are the following:

i) creating the first spatially explicit atlas of oil and gas production and exploration;
ii) estimating the spatial dimension of oil and gas activities and their relationships with ecologically sensitive areas and IPLs.

## 2 Material and methods

### 2.1 Cartographic definition of the Arctic study region

**Fig 1:**
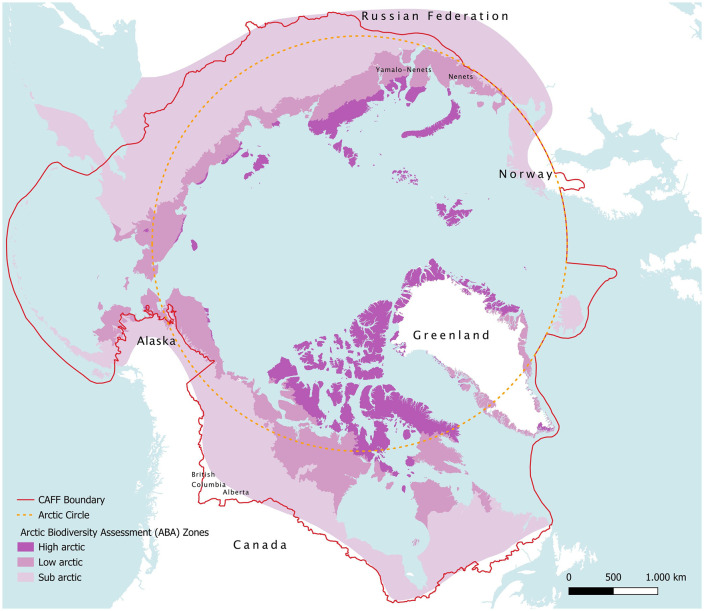
Map of Arctic CAFF study area and comparison with other delimitations. [Basemap source: Global Oceans and Seas by Flanders Marine Institute (2021), licensed under a Creative Commons Attribution 4.0 License. Available online at https://www.marineregions.org/, https://doi.org/10.14284/542].

There are different delimitations of the Arctic area, related to geopolitical or ecological aspects. According to the aim of this study focused on socio-cultural and ecosystems conservation and preservation, the geographic limits adopted to delimit the Arctic region are the boundaries defined by the Conservation of Arctic Flora and Fauna (CAFF) working group of the Arctic Council, which combine climatic and bio-geographical criteria. CAFF boundary is wider than the Arctic Circle and the tree line, including a greater portion of territory, most of all in Canada and Russia. Its total area is around 32.3 million km^2^, of which 57% (18.3 million km^2^) is marine and 43% (14 million km^2^) terrestrial. The rationale behind the use of this boundary is the inclusion of the largest possible portion of vulnerable ecosystems. In fact, the ecological and conservation importance of this area is witnessed by the presence of 92 areas under global international protection policies, among which 12 are World Heritage sites and 80 are Ramsar sites [51]. Cartographic representation of the Arctic CAFF region and the Arctic Circle is presented in Fig 1, where Arctic Biodiversity Assessment (ABA) zones, i.e., the areas investigated by CAFF to assess status and trends of Arctic biodiversity, are highlighted.

Table 1 shows the terrestrial extension of the Arctic countries and their Exclusive Maritime Economic Zones (EEZ) investigated in this study, i.e., Norway, Greenland, Alaska, Russia and Canada. Other CAFF countries, i.e., Iceland, Sweden and Finland, are not participating or planning oil and gas activities. The total terrestrial area considered in this study is about 13.7 million km^2^. i.e., corresponding to about 98% of the terrestrial CAFF region, while the EEZ marine area is about 14.5 million km^2^, or about 79% of total marine Arctic (Table 1).

**Table 1:** Terrestrial and marine surface areas (km^2^) of each case-study country within the CAFF boundary.

	Norway	Greenland	Alaska	Russia	Canada	ARCTIC under study
**CAFF terrestrial** **area (km^2^)**	158,247	2,168,920	615,263	5,402,628	5,380,143	13,725,203
**% out of the CAFF terrestrial area**	1.13	15.48	4.39	38.54	38.38	97.90
**CAFF marine area (km^2^)**	1,643,415	1,736,384	2,178,441	5,009,333	3,899,531	14,467,106
**% out of the total CAFF marine area**	8.93	9.44	11.84	27.22	21.19	78.62

### 2.2 Geospatial data mining and GIS processing

**Fig 2:**
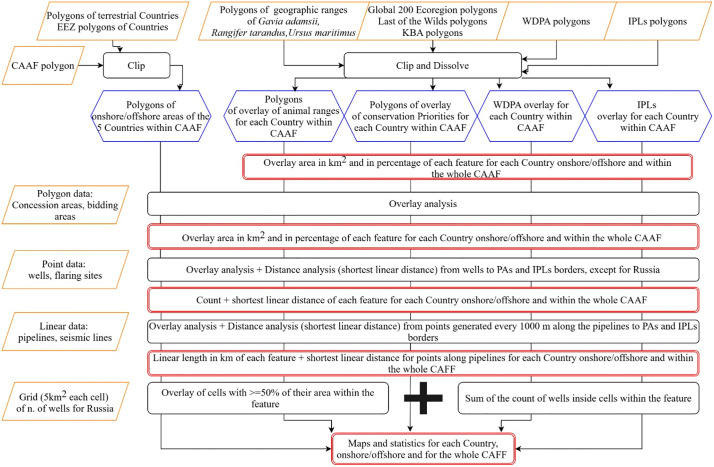
Workflow of methodology. Yellow represents input data, black represents processing, blue intermediate results and red final results.

A workflow (Fig 2) of subsequent but complementary steps was set up to accomplish the aims of this study. First of all, an extensive open spatial data mining was conducted, consulting national and global sources between 2024 and 2025 (except for Greenland’s bidding data). This process enabled the collection of all the geodata available at the time of retrieval, along with their metadata, including data on both active and inactive infrastructure (e.g., inactive wells), which were used in the atlas and can be consulted in the Supporting Information (S1 Table in S1 File).

The data of interest were downloaded and organized in four main geographies (i.e., “boundaries”, “oil and gas”, “ecologically sensitive areas” and “Indigenous Peoples”). The boundary geography includes the limits of the CAFF region and the five countries, and their EEZs under study.

The oil and gas geography regards exploration and production licenses (hereafter referred to as “concessions”), areas under bid, oil and gas infrastructure (including wells and pipelines) and other associated elements such as gas flares and seismic lines. Given the heterogeneity of data collected and the challenges of standardizing licenses under a single legal definition, to clarify the extent and characteristics of the concession and areas under bid datasets, definitions and further descriptions are provided in S6 Table in S1 File. These definitions are based on a review of the areas under bid and concession datasets presented in S1 Table in S1 File and a comparison of their information, described in S2 Appendix in S1 File.

For most countries, the main sources for oil and gas related data were geoportals of national institutions such as oil and gas ministries or energy regulators, whose information was compared and eventually integrated with the Global Oil and Gas Infrastructure (GOGI) database, an effort of the US Department of Energy and its National Energy Technology Laboratory with other partners, to produce a global integrated oil and gas infrastructure open-access database [52], and the Oil and Gas Infrastructure Mapping (OGIM) dataset [53]. It is worth noting that oil and gas features in Russia were estimated mainly using the GOGI dataset, due to difficulties in consulting national websites caused by current international geopolitical issues. This implied some differences for Russia with respect to the other countries: the well dataset is not in the form of a point vector, but as a regular square grid of 5 km side, where each cell contain the estimated absolute number of wells, and concession are not properly areas granted by the government but catalogued as ‘fields’ (areas above a hydrocarbon accumulation) by GOGI. Moreover, there is a lack of data for Russian bidding areas and seismic lines. Data for gas flaring were instead gathered from the Earth Observation Group global database and filtered for each case-study country [54].

The geography of ecologically sensitive areas considered three kinds of areas separately: IUCN natural protected areas (PAs); the geographical ranges of three key Arctic species, i.e., the polar bear (*Ursus maritimus*), the yellow-billed loon (*Gavia adamsii*) and the caribou (*Rangifer tarandus*); and three conservation priorities of NGOs of global importance, such as Global 200 Ecoregions (WWF), Last of the Wild areas, (LW, the Wildlife Conservation Society and Columbia University Center for International Earth Science Information Network), and Key Biodiversity Areas, (KBAs, the KBA Partnership).

The rationale behind the selection of these three species was based on several criteria. First, each species is affected by distinct yet interrelated impact mechanisms associated with oil and gas activities: polar bears may suffer insulation loss, ingestion toxicity, and prey impacts from oil spills overlapping sea-ice habitat [55]; caribou show altered movement and habitat use near energy infrastructure [38,39]; and Arctic seabirds, including the yellow-billed loon, are particularly vulnerable to oil pollution due to feather fouling and disruption of foraging [56,57]. Together, these species represent different Arctic ecosystem compartments (i.e., marine, terrestrial, and avifaunal) providing an integrated perspective on potential environmental impacts. Second, all three species have high conservation relevance and are used to inform environmental assessment and decision-making processes, including spatial planning and impact mitigation [42,58]. Third, their broad ecological requirements allow them to function as umbrella species, capturing potential impacts on wider groups of co-occurring and less-studied species [59,60]. Although not explicitly referred to as “umbrella species” in the literature, the yellow-billed loon functions as one because its conservation protects extensive Arctic tundra and marine habitats vital for many other species, particularly given its reliance on pristine large lakes for breeding and expansive ocean areas for wintering [61]. Finally, all three species hold significant ecological as well as cultural and subsistence importance for Indigenous Peoples across the Arctic [46,47,61–64].

While the IUCN protected areas dataset is widely used in the scientific literature for spatial analyses of oil and gas activities [14,16,65], NGO-led conservation projects were selected based on the layers included in the conservation index developed by the widely used Co$tingNature model [66], which integrates multiple globally recognized biodiversity datasets. Within the CAFF region, three NGO-led conservation layers were ultimately considered in the analysis (namely, Global 200 Ecoregions, LW and KBAs). Further details on dataset selection, overlaps among conservation layers, and methodological considerations are provided in the Supporting Information (S1 Appendix in S1 File).

Lastly, regarding the geography of Indigenous Peoples, we considered the global dataset of IPLs developed by Garnett et al. [67]. In this study, IPLs refer to areas where Indigenous land tenure is formally recognized and where Indigenous communities, according to available data, maintain significant *de facto* influence over land management, defined as “the process of determining the use, development and care of land resources in a manner that fulfils material and non-material cultural needs, including livelihood activities such as hunting, fishing, gathering, resource harvesting, pastoralism and small scale agriculture and horticulture.” ([67], p. 370). This definition was therefore adopted in the present study to delineate IPLs.

It is important to note that the dataset developed by Garnett et al. does not represent a layer describing unopinionated biology but rather a socio-political one, with many of the mapped boundaries being contested. Moreover, as data have been collected from publicly available sources, the extent to which free, prior and informed consent has been obtained cannot be attested. For these reasons, overlaps between IPLs and oil and gas activities cannot be assumed to indicate opposition. Therefore, in the discussion section, some qualitative examples from existing literature are included to qualify overlaps. For further details on data collection methods and limitations, we refer readers to the Supporting Information provided by Garnett et al. [67].

All datasets were then processed in a GIS environment, using the open-source software QGIS and the WGS 84/ Arctic Polar Stereographic (EPSG:3995) as Spatial Reference System. National boundaries and EEZs for each country were clipped within the CAFF to obtain country-specific onshore and offshore CAFF areas, which were then used to select all the other categories included in this region.

Overlay and distance analyses were lastly performed to calculate the spatial interactions among the oil and gas geography and the other geographies and to generate related maps and summary tables. For the overlay analysis, we calculated intersections between the oil and gas features and Arctic onshore/offshore areas, conservation and ecological aspects and IPLs. In the case of Russia, due to the use of a regular grid from the GOGI database to estimate well distribution, only those grid cells with at least 50% of their area overlapping the target polygons were considered. The total well count was then computed based on these selected cells. For the distance analysis, we measured the shortest linear distances between wells, PAs and IPLs, excluding Russia, due to the absence of point-based well data. For pipelines, we generated sampling points every 1,000 meters along the network and calculated the shortest linear distance from each of these points to the nearest PA or IPL. To avoid double counting, duplicate features (e.g., wells or PAs located at the same coordinates) were excluded from the analysis.

## 3 Results

**Fig 3:**
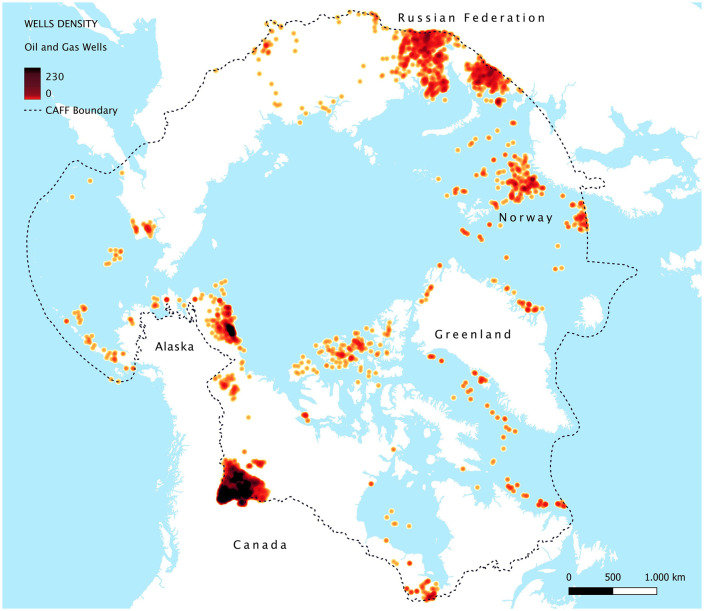
Map of oil and gas well density in the Arctic region. [Basemap source: Global Oceans and Seas by Flanders Marine Institute (2021), licensed under a Creative Commons Attribution 4.0 License. Available online at https://www.marineregions.org/, https://doi.org/10.14284/542].

**Fig 4:**
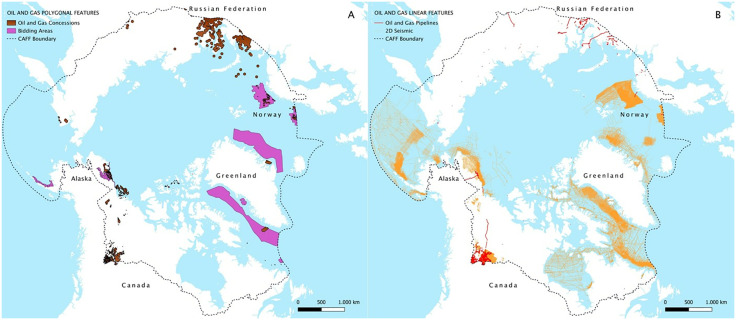
Map of oil and gas polygonal features in the Arctic region (a) and map of oil and gas linear features in the Arctic region(b). [Basemap source: Global Oceans and Seas by Flanders Marine Institute (2021), licensed under a Creative Commons Attribution 4.0 License. Available online at https://www.marineregions.org/, https://doi.org/10.14284/542].

The described workflow allowed the creation of the first Atlas of Arctic oil and gas, starting from many heterogeneous data from different sources, and ending with the maps represented in Figs 3 and 4 of the current management and development of the exploration and extraction activities conducted in the Arctic. Our analysis then focused on the spatial relationships between the geography of oil and gas production and those of ecologically sensitive areas and Indigenous Peoples. The results for both the creation of the Atlas and the spatial analysis between the geographies of interest are displayed in the following section or in the Supporting Information (S2-S5 Tables in S1 File) through quantitative data and cartographies. The results are organized according to the stages of development—seismic surveys, bidding, concessions, wells, pipelines, and flaring—and are distinguished between onshore and offshore data.

### 3.1 The geography of oil and gas production

All the results regarding the dimension of the geography of oil and gas production are shown in Table 2 and Figs 3 and 4. Intense seismic exploration activity has been observed, also in areas without current fossil fuel extraction plans, reflecting the pervasive nature of these activities across the Arctic. Overall, a stunning 1,959,507 km of seismic lines have been calculated throughout the Arctic, that is five times the Earth-Moon distance or almost 48 times the Earth's circumference. Of this total, 338,806 km were traced onshore, while the majority was conducted offshore (1,620,701 km).

**Table 2:** Spatial relationships between oil and gas geographies and the Arctic region.

Features	Norway	Greenland	Alaska	Russia	Canada	ARCTIC
**Concessions (onshore) (km**^**2**^)	0	8,306	18,515	308,965	68,487	404,273
% in the total CAFF onshore area of each country	0.00%	0.38%	3.01%	5.72%	1.27%	2.89%
**Concessions (offshore) (km**^**2**^)	24,863	10,061	2,848	45,271	24,990	108,033
% in the total CAFF offshore area of each country	1.51%	0.58%	0.13%	0.90%	0.64%	0.75%
**Concessions (onshore + offshore) (km**^**2**^)	24,863	18,367	21,363	354,236	93,477	512,306
% in the total CAFF area of each country	1.38%	0.47%	0.76%	3.40%	1.01%	1.82%
% of onshore concessions in the total Arctic concessions	0.00%	45.22%	86.67%	87.22%	73.27%	78.91%
% of offshore concessions in the total Arctic concessions	100.00%	54.78%	13.33%	12.78%	26.73%	21.09%
% of total concessions (onshore + offshore) in total Arctic concessions	4.85%	3.59%	4.17%	69.15%	18.25%	100.00%
**Area open for bidding (onshore) (km^2^)**	0	12,706	45,807	no data	0	58,513
**Area open for bidding (offshore) (km^2^)**	184,174	826,514	10,888	no data	6,062	1,027,638
**Area open for bidding (onshore + offshore) (km**^**2**^)	184,174	839,220	56,695	no data	6,062	1,086,151
% on CAFF area of each country	10.22%	21.49%	2.03%	no data	0.07%	3.85%
% of the area open for bidding in total Arctic area open for bidding	16.96%	77.27%	5.22%	no data	0.56%	100%
**Onshore wells (num.)**	0	90	7,844	2,699	32,801	43,434
% out of total onshore wells	0.00%	0.21%	18.05%	6.29%	75.46%	100.00%
**Offshore wells**	577	43	321	34	130	1,105
% out of total offshore wells	52.57%	3.88%	28.94%	2.89%	11.72%	100.00%
**Total wells (onshore + offshore)**	577	133	8,165	2,733	32,931	44,539
**Gas flares**	1	0	25	439	47	512
% out of total flares	0.20%	0.00%	4.88%	85.74%	9.18%	100.00%
**Onshore pipelines (km)**	0	0	1,225	6,580	31,440	39,245
**Offshore pipelines (km)**	200	0	8	82	0	290
**Total pipelines (km)**	200	0	1,233	6,662	31,440	39,535
% on total CAFF pipelines length	0.51%	0.00%	3.12%	16.85%	79.52%	100.00%
**Onshore seismic lines (km)**	0	1,858	23,649	no data	313,299	338,806
**Offshore seismic lines (km)**	525,290	319,880	309,382	no data	466,149	1,620,701
**Seismic lines (km)**	525,290	321,738	333,031	no data	779,448	1,959,507
% of seismic lines out of total seismic lines	26.81%	16.42%	17.00%	0.00%	39.78%	100.00%

Most onshore seismic prospecting within the CAFF region has occurred in Canada (313,299 km), primarily in Nunavut, where extractive activity has largely been associated with exploration, as well as in the provinces of British Columbia and Alberta. Onshore seismic lines are also present in Alaska (23,649 km) and Greenland (1,858 km).

Regarding offshore seismic prospecting, Norway’s maritime areas—particularly the Barents Sea—account for 525,290 km of 2D seismic lines, while offshore Greenland contributes 321,738 km (Fig 3). In Canada, large activity has been observed offshore within the Hudson Bay and Labrador Sea. Despite existing moratoria on offshore extraction in the Arctic region such as those in Canadian and Alaskan Arctic waters [20], numerous past seismic lines are visible in the available data, with many of these that were traced before the current moratoria were put in place. Regions without current production but with a widespread presence of these lines include the eastern and western offshore areas of Greenland, Norwegian Svalbard, and northern Canada (Fig 3).

We could not obtain any reliable information on 2D seismic lines in Russia, despite the region’s well-documented history of exploratory activities [68], partially affecting our spatial analyses. Nevertheless, considering other countries’ data, the cumulative length of seismic lines across the Arctic confirms the already mentioned intense activity of exploration in the region.

Arctic bidding areas cover a total of 1,086,151 km^2^—an area comparable to the surface of Bolivia—representing 3.85% of the Arctic region and more than double the extent of concessions. Only a small portion of these areas is located onshore, in Alaska (45,807 km^2^) and Greenland (12,706 km^2^). Most Arctic bidding areas are offshore, owned by Greenland (77.27% of total bidding areas within the CAFF region), comprising 21.49% of the country’s total Arctic territory. Moreover, the spatial analysis identified several areas that were available for bidding in Canada, particularly in the Atlantic region, specifically offshore of the southern Labrador province, the most recent in 2024 [69]. However, only a limited part of these areas falls within the CAFF boundary, consequently excluding the majority from our analysis. As the case for 2D seismic, no accessible information was found for bidding areas in Russia.

512,306 km^2^ of the Arctic region are covered by concessions, representing 1.93% of the study area, a territory size comparable to that of Spain or Thailand. The extraction activity concentrates mostly on land (78.91% of the surface of total concessions) rather than in waters. With extensive coverage, the majority of oil and gas territories are located in Russia, occupying 354,236 km^2^, equivalent to 69.15% of the total Arctic concessions. Russia is then followed by Canada (18.2%), Norway (4.85%), Alaska (4.17%) and Greenland (3.59%). Russia also holds the record for the largest portion of onshore extraction area in the Arctic, covering 308,965 km^2^, nearly 6% of Russia’s Arctic land. In the Russian Arctic, extraction activity is mostly concentrated in the West Siberia petroleum basin within the Yamalo-Nenets and the Nenets Autonomous Okrug.

Although Alaska’s concession area is relatively smaller compared to other Arctic countries, it allocates around 3% of its land territory to concessions. Leases in Alaska are mostly concentrated in the North Slope (northern Alaska), especially within the onshore areas of Prudhoe Bay and the National Petroleum Reserve with the latter experiencing a rapid expansion of oil and gas activities in the last two decades [70].

Regarding the offshore concessions, Russia holds the largest (45,271 km^2^), with upstream activities primarily occurring in the Pechora, Barents and Kara Seas. Canada’s offshore concessions (24,990 km^2^ are primarily concentrated in the Atlantic region, in the Labrador Shelf. Additional exploration licenses exist in the Arctic Beaufort Sea, off the coasts of the Northwest Territories and Nunavut, as well as in parts of the Arctic Archipelago, though these licenses are limited and largely inactive due to the federal moratorium on new oil and gas activities in Canadian Arctic waters north of 60° latitude [20]. Norway’s concessions are located in the Norwegian Sea and, to the north, in the Barents Sea, covering 24,863 km^2^, which represents 1.38% of the country’s EEZ in the Arctic region. Finally, Greenland holds 10,061 km^2^ offshore licenses exclusively dedicated to oil exploration.

Oil and gas wells are unequally distributed across the Arctic region, with Canada presenting the largest number of onshore wells (32,801), which accounts for 75.46% of the total amount of onshore wells. While Canada presents the highest number of wells, Greenland has the fewest, with a total of 143, due to its reduced extraction activity, in favour of the exploration one, conducted by seismic studies. The majority of offshore wells are distributed between Alaska (321) and Norway (577), corresponding to the 28.94% and the 52.57% respectively of the total offshore wells in the Arctic. For Russia, a total of 2,733 wells has been estimated; however, it is not possible to rely with high confidence on this number given the different nature of the data used, as explained in section 2.2.

Gas flaring data indicate that Russia is the largest contributor to gas flaring in the Arctic, with 439 gas flaring activities detected in correspondence ofconcessions for the year 2022 (85.74% of the total Arctic flaring activities). The Russian government attempted to mitigate gas flaring through Decree No. 1148 of 2012, which aims to limit the amount of gas flared or vented to 5% of the total volume of associated gas. Despite this legislative effort, the prevalence of gas flaring sources remains significant compared to other Arctic countries. In contrast, flaring sites are absent in Greenland and present much lower values in other study countries.

Regarding linear elements, our analysis revealed that approximately 39,535 km of pipelines are spread across the Arctic Region, a measure more than six times the Earth radius. Specifically, terrestrial pipelines cover about 39,245 km, whereas offshore pipelines constitute a smaller portion (290 km). The linear development of the pipelines is extensive and predominant in Canada, with a dense network of 31,440 km pipelines able to move hydrocarbons through the vastness of its territory, followed by Russia, accounting for 6,662 km. Alaska presents 1,233 km, with the largest contribution coming from the Trans-Alaska Oil Pipeline System, accounting for about 400 km within the CAFF Arctic. Greenland presents no pipelines on its land nor on its maritime areas.

### 3.2 Spatial overlaps and interactions between the geography of oil & gas production, ecologically sensitive areas and Indigenous Peoples’ lands

#### 3.2.1 Ecologically sensitive areas.

**Fig 5:**
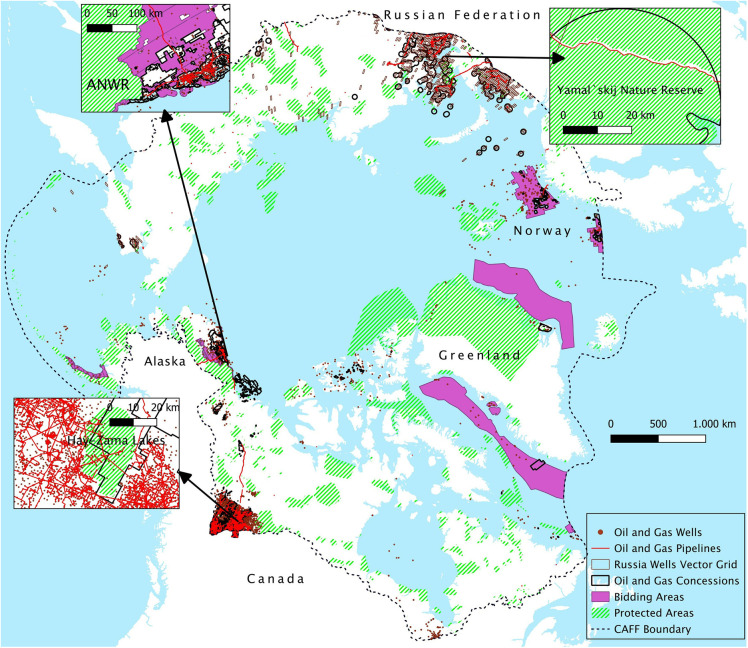
Map of oil and gas features and PAs in the Arctic region. [Basemap source: Global Oceans and Seas by Flanders Marine Institute (2021), licensed under a Creative Commons Attribution 4.0 License. Available online at https://www.marineregions.org/, https://doi.org/10.14284/542].

Across the entire study area, approximately 39,000 km^2^ of the total surface area of PAs in the Arctic overlaps with concessions, which constitutes a small portion when compared to the overall extent of Arctic PAs (3,721,653 km^2^) (S2 Table in S1 File, and Fig 5). However, when considering the percentage of concessions overlapping with PAs, we found that over 7% of these concessions intersect with PAs. The largest contribution comes from Russia, covering 34,554 km^2^.

Spatial overlaps between hydrocarbon bidding areas and PAs are generally limited (2,441 km^2^ across the entire CAFF region), with the exception of Alaska, where 1,542 km^2^ are available for lease sale within PAs. Regarding the presence of oil and gas wells in PAs, 403 drilled wells have been identified, corresponding to 0.90% of the total wells in the Arctic, with Canada (214) and Russia (125) being the largest contributors in absolute terms. The GIS analysis also showed the presence of about 382 km of pipelines within Arctic PAs (0.97% of total pipelines), distributed between Canada (309 km) and Russia (73 km). Notable cases of PAs affected by wells and pipelines disturbance are the Hay-Zama Lakes Wildland Provincial Park in Alberta province (IUCN category Ib) (Fig 5) and the National Parken in Greenland.

**Fig 6:**
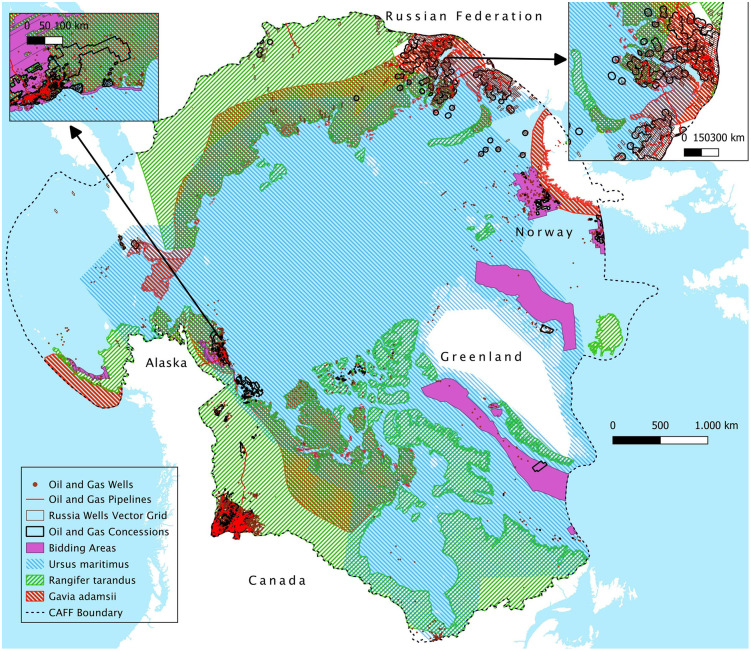
Map of oil and gas features and species ranges in the Arctic region. [Basemap source: Global Oceans and Seas by Flanders Marine Institute (2021), licensed under a Creative Commons Attribution 4.0 License. Available online at https://www.marineregions.org/, https://doi.org/10.14284/542].

Although the ranges of species only marginally overlap with oil and gas activities compared to their huge geographic extent across the entire Arctic, exploratory and production activities must consider the potential presence of these species. In fact, spatial analysis reveals that 50.56%, 42.38%, and 30.42% of the concessions coincide with the geographic ranges of *Ursus maritimus, Gavia adamsii, and Rangifer tarandus*, respectively (S3 Table in S1 File, and Fig 6). Specifically, the majority of Alaskan leases is overlapped by the ranges of *Gavia adamsii* (87.45%), *Rangifer tarandus* (87.21%), and *Ursus maritimus* (100%) (Fig 6). Similarly, the geographic range of *Ursus maritimus* overlaps all licenses in Greenland, with this species being central to CAFF region bidding areas, as 87.30% of these areas overlap its range. Furthermore, over 73% of Canadian concessions are overlapped by the range of *Rangifer tarandus*. Overall, 87.21% of leases in Alaska and over 13% of total Arctic concessions are overlapped by the ranges of all three species, highlighting the urgent need for ecological conservation-oriented spatial planning in oil and gas concession areas, particularly in Alaska [47,71].

Similar trends are observed in the overlap analysis with physical infrastructures. Approximately 1,618 km of pipelines (4.09% of the total) intersect the ranges of all three species, with Alaska being the largest contributor (1,065 km). Additionally, three priority areas are also overlapped by 7,701 wells (17.29% of the total), with the majority (7,400) located in Alaska.

**Fig 7:**
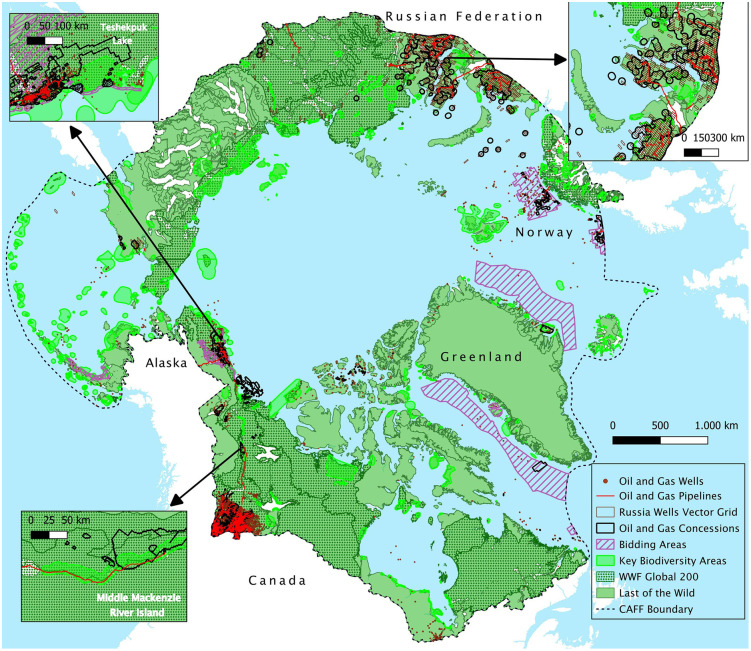
Map of oil and gas features and NGO-conservation priorities in the Arctic region. [Basemap source: Global Oceans and Seas by Flanders Marine Institute (2021), licensed under a Creative Commons Attribution 4.0 License. Available online at https://www.marineregions.org/, https://doi.org/10.14284/542].

Arctic concessions overlap with KBAs by 5.25%, LW areas by 71.66%, and Global 200 ecoregions by 15.74%. In Alaska, where existing production facilities and future development prospects are of particular concern, 12.55% of leases intersect with KBAs (S4 Table in S1 File, and Fig 7). Of particular concern is the Teshekpuk Lake – E. Dease Inlet, renowned for its ecological sensitivity due to its rich biodiversity and wetland ecosystems. Although this area presents limited overlaps with leases (3.48%), it is encircled by leases (Fig 7). This concern is amplified by the significant overlap with the other two conservation areas. Additional areas of concern include the Coville River Delta and the Beaufort Sea nearshore. The latter accounts for 261 wells inside its surface.

Alaska also highlights a critical spatial conflict between these conservation priorities and bidding areas, with 23.10%, 67.34%, and 46.10% of bidding areas overlapping with KBAs, LW, and WWF ecoregions, respectively. 60 wells have been drilled in areas overlapping the three priorities, with the majority located in the Teshekpuk Lake KBA.

In Canada, approximately 1.39% of concessions coincide with the three overlapping conservation priorities. These areas include portions of KBAs, such as the Mackenzie River Delta. This result highlights the importance of the moratorium on oil and gas drilling in Arctic waters for conservation efforts, as the Mackenzie River Delta KBA is overlapped by 9.51% by licenses included in the moratorium. Other KBAs affected by concessions include the lower and middle Mackenzie River Island KBAs and Hay Zama Lakes (Fig 7). It is worth noting that within these areas, oil production is currently only taking place in the middle Mackenzie River Island KBA, in Norman Wells extraction sites. Additionally, the Middle Mackenzie River Island KBA is intersected by part of the Norman Wells Oil Pipeline (Fig 7), which constitutes 320 km of the 321 km of pipelines crossing the three overlapping conservation priorities in the CAFF region. Regarding Russia, approximately 5% of extraction areas are situated within KBAs. Finally, Norway does not exhibit spatial overlaps between the three conservation priorities and oil and gas spatial planning.

#### 3.2.2 Indigenous Peoples’ lands.

**Fig 8:**
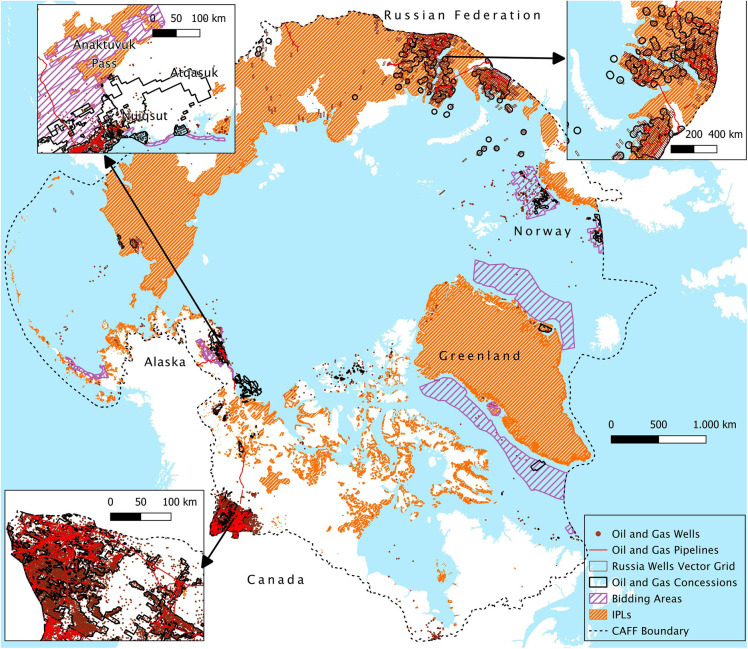
Map of oil and gas features and IPLs in the Arctic region. [Basemap source: Global Oceans and Seas by Flanders Marine Institute (2021), licensed under a Creative Commons Attribution 4.0 License. Available online at https://www.marineregions.org/, https://doi.org/10.14284/542**].**

Oil & gas concessions cover more than 4% of IPLs in the Arctic, while 73.3% of all Arctic oil and gas concessions are located within IPLs (S5 Table in S1 File, and Fig 8). In Russia, over 92% of concessions coincide with IPLs, for a total of 285,085 km, an area comparable to Ecuador. This surface is equal to 6.59% of the total IPLs in Russia. Only 0.38% of IPLs in Alaska overlap with oil & gas leases, and only 2% of leases coincide with IPLs. Despite this limited overlap, we observed that several community settlements are located border to border to leased territories in the North Slope, but with no or limited overlap (Fig 8).

A total of 3,146 drilled wells has been identified within IPLs across the Arctic, corresponding to 7,24% of the total wells in the region. The largest contribution comes from Russia with 2,428 wells, followed by Alaska (429), Canada (203) and Greenland (86). The data for Canada is surprisingly low if compared to the dimension of the geography of fossil fuel production presented in the previous paragraphs. The relatively low overlap between oil and gas infrastructure and IPLs can be attributed to the limited extent of IPLs within British Columbia and Alberta (891 km^2^, less than 0.2% of Canada’s IPLs), which are the provinces with the highest density of wells in the country. Around 5,934 km of pipeline cross IPLs in the Arctic region, with over 5,500 km located in Western Siberia (around 84% of total Russian pipelines), 384 km in Canada and 34 km in Alaska.

The results for Norway and Greenland deserve a separate mention. For instance, Norway’s oil and gas activities are exclusively offshore, thus avoiding spatial overlap with IPLs. Conversely, in Greenland, all onshore land is considered Indigenous, with nearly 100% of concessions overlapped by IPLs. However, only 0.39% of this land is overlapped by concessions. Furthermore, the current halt to oil and gas activity in Greenland significantly reduces the spatial overlap between potential industrial interests and Indigenous communities.

#### 3.2.3 Distance of wells and pipelines to protected areas and Indigenous Peoples’ lands.

Table 3 presents the shortest linear distance between oil and gas infrastructure—specifically pipelines and wells—and the borders of PAs and IPLs across the Arctic countries under study. The “Total” rows refer to the full set of wells and to the total points generated at 1,000-meter intervals along the pipeline network located outside PAs and IPLs. This analysis includes both onshore and offshore developments collectively. Distances are presented as the percentage of features falling within 1,000-meter intervals, up to 5,000 meters, as well as the share beyond 5,000 meters.

**Table 3:** Shortest linear distance from oil and gas wells and pipelines to the borders of PAs and IPLs.

Distance pipelines to PAs	Canada	Alaska	Russia	Norway	Greenland
**Total outside PAs (points)**	32,735.00	1,238.00	6,666.00	200.00	0.00
**<1000 m (%)**	0.35	0.32	1.49	0.00	0.00
**1000-1999 m (%)**	0.53	0.73	0.33	0.00	0.00
**2000-2999 m (%)**	0.49	2.02	0.36	0.00	0.00
**3000-3999 m (%)**	0.70	2.58	0.26	0.00	0.00
**4000-5000 m (%)**	0.84	2.18	0.26	0.00	0.00
**>5000 m (%)**	97.08	92.16	97.31	100.00	0.00
**Distance wells to PAs**	**Canada**	**Alaska**	**Russia**	**Norway**	**Greenland**
**Total outside PAs (count)**	32,068.00	6,209.00	no data	531.00	72.00
**<1000 m (%)**	0.32	0.11	no data	0.00	1.39
**1000-1999 m (%)**	0.54	0.13	no data	0.19	2.78
**2000-2999 m (%)**	0.58	0.08	no data	0.38	0.00
**3000-3999 m (%)**	0.76	0.13	no data	0.19	6.94
**4000-5000 m (%)**	0.95	0.08	no data	0.38	0.00
**>5000 m (%)**	96.86	99.47	no data	98.87	88.89
**Distance pipes to IPLs**	**Canada**	**Alaska**	**Russia**	**Norway**	**Greenland**
**Total outside IPLs (points)**	32,599.00	1,256.00	1,150.00	201.00	7.00
**<1000 m (%)**	1.18	0.56	2.70	0.50	0.00
**1000-1999 m (%)**	1.11	0.32	1.48	1.00	0.00
**2000-2999 m (%)**	1.11	1.04	1.30	2.99	0.00
**3000-3999 m (%)**	1.21	0.64	1.04	5.47	0.00
**4000-5000 m (%)**	1.02	0.24	1.39	2.99	0.00
**>5000 m (%)**	94.36	97.21	92.09	87.06	100.00
**Distance wells to IPLs**	**Canada**	**Alaska**	**Russia**	**Norway**	**Greenland**
**Total outside IPLs (count)**	**32,052.00**	**5,860.00**	**no data**	**537.00**	**31.00**
**<1000 m (%)**	0.38	0.48	no data	0.00	12.90
**1000-1999 m (%)**	0.46	0.26	no data	0.00	0.00
**2000-2999 m (%)**	0.52	1.74	no data	0.00	0.00
**3000-3999 m (%)**	0.48	0.27	no data	0.00	0.00
**4000-5000 m (%)**	0.58	0.14	no data	0.00	0.00
**>5000 m (%)**	97.59	97.12	no data	100.00	87.10

As already discussed in earlier sections, Canada, Russia, and Alaska host the majority of mapped oil and gas infrastructure. In general, between 87% and 99% of wells and pipeline-related points lie more than 5 km away from PAs and IPLs. The highest proportion of pipeline sampling points within 1 km of a PA is found in Russia (1.49%), increasing to 2.69% within 5 km. Canada and Alaska show greater overall proximity within the 5 km threshold, with 2.92% and 7.83% of points, respectively. For wells, Greenland exhibits the highest relative proximity to PAs, with over 11% of its (albeit few) 72 wells located within 5 km. Canada follows with approximately 3%, corresponding to over 1,000 wells. Russia was excluded from well-distance calculations due to the grid-based format of its data.

Proximity to IPLs is more pronounced than to PAs. For pipeline sampling points, Canada and Russia report 1.18% and 2.70% of points within 1 km of IPLs, increasing to 5.63% and 7.91% within 5 km, respectively. Norway shows the highest 5 km interaction rate, with nearly 13% of pipeline points located within that range. Regarding well proximity to IPLs, Greenland again leads with 12.90% of its 31 wells falling within 1 km.

## 4 Discussion

### 4.1 Overlap and distance analysis

The spatial analysis conducted in this study allowed us to map the geographies of the overlapping interests between hydrocarbon production and ecological and Indigenous Peoples spatial priorities across the Arctic’s main fossil fuel basins. In particular, findings from CAFF Canada reveal a high density of oil and gas infrastructure in the provinces of Alberta and British Columbia, far exceeding the extractive presence in other Arctic concession areas. These regions are emblematic of how extensive onshore extractive activities, including oil and gas wells and seismic exploration, have dramatically altered boreal and tundra ecosystems, contributing to habitat fragmentation, edge effects, and the proliferation of road networks as well as impacting Indigenous Peoples’ quality of life [36,37,72]. Although seismic impacts are often associated with terrestrial environments, our results reinforce growing concerns regarding their offshore implications. While a specific analysis of the impacts of seismic exploration was not conducted in this study, our findings corroborate the concerns raised by Reeves et al. [41] regarding the potential impacts of induced seismic activity on marine fauna, particularly cetaceans.

For what concerns IPLs, the results provide a picture of spatial interactions between Indigenous communities and the prospects of development for oil and gas Arctic basins, particularly in Russia’s Yamal Peninsula and Alaska’s North Slope. These interactions have been extensively documented, particularly in the Yamal Peninsula, where the nomadic Nenets people are affected by the impacts of oil and gas activities and infrastructure on their reindeer migration routes, crucial for their livelihood [46,73].

The interactions between extraction activities and various priorities extend beyond spatial overlaps, as emerged in the shortest distance analysis from oil and gas infrastructures to PAs and IPLs. Therefore, a combination of overlay analysis coupled with proximity analysis could provide a more effective depiction of the real situation and strengthen the criteria used in multicriteria evaluations, which could be explored in future research.

Focusing on the shortest distance between oil and gas wells and pipelines at local scale in proximity to PAs reveals that in some cases borders were cut or delimited in a way that avoid overlaps with oil and gas activities, or vice versa, reflecting the limitations of these protection policies. The Bovanenkovo-Ukhta Gas Pipeline in Russia is an infrastructure related to this phenomenon. Despite passing across the Yamal`skij Nature Reserve (established in 1977), this pipeline shows a very limited overlap with the PA, since the reserve has been adjusted in correspondence with the pipeline's route (Fig 5) [74]. These findings corroborate those of other authors concerning the downsizing or redesign of PAs to provide space for economic activities [75–77]. Regarding this phenomenon, the LINGO initiative [78] also highlighted the potential future consequences of the 2022 law adopted by the State Duma allowing for changes in the boundaries of Specially Protected Natural Areas.

Oil and gas elements within 5 km distance can be observed for the Arctic National Wildlife Refuge in Alaska (ANWR) (Fig 5) or the Maxhamish Lake Protected Area in Canada. In Alaska, only 0.38% of IPLs overlap with oil and gas leases and only 2% of leases coincide with IPLs. Despite this limited overlap, we observed that several community settlements are located border to border to leased territories in the North Slope but with no or limited overlap (Fig 8). This is the case, for example, of the Nuiqsut community, surrounded by leases related to the recent Willow project. Indeed, in the final Supplemental Environmental Impact Statement for the Willow project released in February 2023, it is predicted that the Nuiqsut population will be hugely affected by the project’s impacts on their livelihood [79]. These impacts are expected to also extend to other Native American communities such as Anaktuvuk Pass and Atqasuk [79]. The impacts deriving from the Willow project would add to the already well-documented spatial conflicts between oil and gas extraction and several communities in the North Slope [47,79].

While it is important to acknowledge that several Indigenous communities engage in various forms of collaboration with the oil and gas industry and extractive activities (as discussed in Section 1), this discussion focused on highlighting the areas of existing or potential conflict faced by communities that are excluded from oil and gas development, thus prioritizing these areas for ceasing extraction and leaving fossil fuels underground. The spatial overlaps presented in this study do not necessarily imply current oil and gas activity in ecologically sensitive areas or IPLs, but rather reflect cumulative and regulatory exposure over time (as explained in detail in S2 Appendix in S1 File). Together, these results suggest that the absence of formal overlap between IPLs or ecologically sensitive areas and the oil and gas geography does not preclude significant socio-ecological impacts. Indeed, the proximity of industrial projects to these geographies can cause cumulative physical, socio-cultural, and economic pressures, threatening human and environmental well-being. In this light, the findings emphasize the importance of integrating spatial justice considerations into Arctic energy governance, including Indigenous consultation, social impact assessment and buffer zones from extraction activities.

### 4.2 Oil and gas data challenges and the case of Russia

Country-wise data mining highlighted how much the degree of data accessibility, typology and accuracy varies across the different countries of the region (further details are provided in S1 File). Firstly, while certain countries provide spatial data through geoportals and consistently updated public data—of which the most glaring example is Norway—others, such as Russia, lack comparable levels of transparency and sensitivity. Countries that have a high level of regionalization, such as Canada, suffer from data heterogeneity among provinces and territories, while having relevant data availability. Abundance of data is not always an advantage, especially in case of similar data from different sources or data with incomplete metadata or attribute tables. In general, the most effective approach to comprehending the data has been through comparison and cross-referencing.

Secondly, another limitation is related to the diversity in data representation concerning the spatial planning of oil and gas extraction across different countries (as described in S2 Appendix in S1 File). Throughout this study, the term 'concession' was used as a geographical term to denote the different kinds of oil and gas spatial planning in Arctic countries. While data used for Alaska, Canada, Greenland and Norway were sourced from institutional channels, providing an official spatial depiction of oil and gas concessions allocated by national authorities to respective companies, data for oil and gas licenses in Russia were not accessible through Russian institutional sources. Consequently, they were acquired from the GOGI global database. Note that a similar approach was adopted for IPLs and ecologically sensitive areas, for which globally available datasets were used to ensure consistency across Arctic countries and to avoid problems related to uneven availability of national or sub-national data.

To our knowledge, national data on licenses in Russia are only accessible for visualization through portals such as those provided by Rosnedra and Rosneft. The unavailability of downloadable data for licenses reflects a broader restrictive policy concerning sensitive information in the country and, following the Russian invasion of Ukraine, access to sensible data like those on oil and gas, environmental protections and Indigenous Peoples may have faced further restrictions.

While we recognize that obtaining more reliable and up-to-date data could have been achieved through commercial sources, we believe that research must be open and reproducible. For this reason, we opted for an approach based on open and freely accessible data gathered bottom-up, from country sources. As of now and despite the valuable work of GOGI and OGIM, a comprehensive global open dataset on concessions, infrastructures and reserves remains unavailable to the scientific community. We firmly advocate for the public availability of such data to empower the scientific community in conducting studies that can inform policymaking regarding the fossil fuel phase-out and leaving fossil fuels underground.

Despite the limitations included in our data for concessions (S2 Appendix in S1 File), they serve a valuable purpose in mapping out the spatial intersections between the geographies representing the various stakeholders and interests (e.g., industrial economic development versus conservation), as already conceptualized, for example, by Cuba et al. [65] and Codato et al. [16]. Indeed, the “concessions” used in this study represent areas where extractive activity occurred, is occurring or could occur in future (S6 Table in S1 File). As such, these concessions reflect geographies of possible perturbations in socio-environmental development patterns and, therefore, potential spatial overlaps between the claims of extractive industry and conservation and Indigenous Peoples instances.

### 4.3 Further geographical investigations for the Arctic region

Additional challenges arise when attempting to define potential metrics for buffer areas to delineate the territorial extents of nomadic and semi-nomadic Indigenous populations in the Arctic region as it is not possible to generalize their behaviour into a common metric since the extent of their migrations could vary significantly among the different groups. Despite not entirely overcoming these limitations, the GIS dataset developed by Garnett et al. [67] was used, currently being, to our knowledge, the most comprehensive dataset for representing the global distribution of territories under control of Indigenous Peoples.

Additionally, since Indigenous communities’ perspectives on oil and gas development cannot be generalized (as noted in Section 1), we believe that the production of a global comprehensive mapping on the issue of Indigenous Peoples' free prior informed consent according to the United Nations Declaration on the Rights of Indigenous Peoples [80], would be extremely useful to truly understand the entity of the overlaps between IPLs and extraction activities, thereby contributing to the identification of areas where oil and gas extraction should be halted, in accordance with the principle outlined by Muttitt and Kartha [50] of stopping extraction where it violates human rights.

Regarding the ecologically sensitive areas, we recognize that the choice for the three target species, given their terrestrial character, may overlook important aspects concerning the spatial conflicts between oil and gas offshore extraction and Arctic fauna. However, the methodology proposed in this study can serve as a starting point to be replicated in future works. For example, an important research would be the one focusing on spatial interactions between oil and gas extraction and the different clusters of species at the basis of the livelihoods of Arctic Indigenous groups.

Using the CAFF boundary to delimit the Arctic region allowed us to include in this analysis parts of two key fossil fuels–producing provinces in the world (British Columbia and Alberta) that would not have been otherwise considered if we had used another boundary (e.g., the Arctic Circle). This inclusion allowed us to add complexity to the study and better understand the dimensions of fossil fuel extraction in the CAFF region. In addition, since the extraction activities are generally concentrated in specific regions within countries, downscale analyses on the local dimension are needed in future research. We believe that a detailed focus within countries is necessary to gain a more comprehensive understanding of the spatial interactions between various geographies of interest in the Arctic. Employing a finer-grained scale could facilitate more detailed and specific analysis regarding oil and gas extraction impacts on biological and cultural priorities in producing regions. It could also allow for the development of potential unburnable carbon scenarios aimed at fossil fuel extraction phase-out based on spatial justice criteria, using spatial multi-criteria decision analysis, as suggested by Codato et al. [19]. In this regard, we have identified the CAFF oil and gas producing provinces of British Columbia and Alberta in Canada, the Yamalo-Nenets and the Nenets Autonomous Okrugs in Russia and the North Slope in Alaska as key study areas for such further analysis.

Lastly, further geographical investigations for the Arctic region could build upon the systematic assessment presented in S2 Appendix of the S1 File, which documents the availability and completeness of temporal and categorical information for wells, bidding areas, and concessions across Arctic jurisdictions. While the assessment indicates that fully harmonized temporal overlap analyses are not feasible at the Arctic scale due to heterogeneity in data structures, licence types, and temporal semantics, it provides a transparent foundation for future regional or country-specific investigations that explicitly consider whether a well or licence were active and the timing of their approval relative to the establishment of PAs or IPLs. Such analyses could support more precise temporal assessments of overlaps and their nature.

### 4.4 Pathways for unburnable carbon policies: Towards an Arctic fossil fuel non-proliferation zone

One of the key issues emerging from this study is the temporal reliability of data and its susceptibility to shifts in national and international policy directions, which can significantly affect decisions on the ground. For example, our findings for bidding areas are influenced by the inclusion of Greenland’s bidding areas, despite the Greenland government’s decision to stop issuing new oil and gas exploration licenses, which represents one of the most remarkable cases of the unpredictability of policy directions [21,81,82].

Another notable example is the moratorium on Arctic waters adopted jointly by Canada and the U.S. [20]. The reactivation of several licenses depends on this policy. Similar issues can be found in relation to the exploration in the ANWR in Alaska, first issued by the Trump administration and then cancelled by the Biden administration in 2023 [70].

At the international level, global policies and practices of environmental lobbying might influence national choices and on the impact of the petroleum industry, above all the UNFCCC and IPCC studies on climate change. For example, the recent COP 28 Global Stocktake introduced the concept of “transitioning away” from fossil fuels in the UNFCCC discussion for the first time [2]. In parallel, supply-side initiatives aimed at promoting the development of a “Fossil Fuel Non-Proliferation Treaty” are gaining increasing consideration (Newell and Simms, 2020) and coalition of early movers are forming, as the case of the Beyond Oil and Gas Alliance, of which Greenland is already a member [83].

While recent national and international initiatives may suggest momentum toward phasing out oil and gas development in the Arctic, the current geopolitical and policy landscape reveals significant contradictions. Despite growing global awareness of the need for climate action, no Arctic or observer state is effectively leading a just transition in the region [84]. The absence of a clear frontrunner reflects a deeper structural problem: fragmented leadership, entrenched fossil fuel dependency, and limited procedural justice mechanisms.

McCauley et al. [84] highlight that Nordic and European countries, such as Sweden, Denmark, Finland, and Iceland, are best positioned to provide normative leadership due to their low fossil fuel dependency, strong governance frameworks, and commitment to procedural and restorative justice. Yet, even these relatively progressive states fall short of the transformative leadership needed to address the full scale of Arctic climate and equity challenges. Their contributions to climate finance remain modest, and their influence is constrained by geopolitical dynamics beyond their control.

At the same time, historically high-emitting countries and large producers such as the United States and Canada, while morally obligated to lead due to their past emissions and greater economic capacity [10,11], are failing to do so in practice. These states perform poorly across all three justice dimensions, distributional, procedural, and restorative.

Moreover, climate justice efforts in the Arctic are undermined by the role of the Russian federation, which overwhelmingly dominates Arctic fossil fuel production, accounting for over 91% of the region’s output in 2022. Indeed, about 80% of Russian gas and 60% of its oil originate from Arctic territories [31]. Even without exploiting new reserves, current production levels alone would exceed a Paris-aligned 1.5°C carbon budget by 200–300% by 2050, requiring the decommissioning of roughly half of Russia’s active Arctic fields to stay on track [31]. However, Russia does not align with international climate justice principles and has shown no meaningful engagement with multilateral sustainability frameworks, making it a structural obstacle to any coordinated and justice-based fossil fuel exit [84].

The unstable geopolitical context further complicates the picture. Following the invasion of Ukraine, the EU has banned most of the import of Russian oil. In response, Russia has shifted its fossil fuel exports towards Asian markets, especially China and India, perpetuating the dependency on oil and gas exports [85]. Consequently, Norway is expected to assume the role previously held by Russia as the primary exporter to the EU by expanding its production in the Barents Sea [31]. In North America, projects like the Willow development in Alaska and the ongoing extraction of Canada’s highly emissive oil sands further exacerbate the divergence from the 1.5°C-aligned pathway in the energy sector [31,79].

In the absence of a legally binding international treaty to leave fossil fuels in the ground, SEI et al [86] estimates that current Arctic production trends remain fundamentally incompatible with the emissions pathways required by the Paris Agreement. As McCauley et al. [84] argue, without enforceable climate commitments and clear leadership, the Arctic risks becoming a climate justice blind spot where symbolic action replaces substantive progress. Cassotta and Mazza [48] reinforce this view by highlighting the fragmented nature of Arctic environmental governance and the systemic failure to meaningfully include Indigenous rights and social impact assessments in fossil fuel regulation. Their work underlines the urgent need for a new legal paradigm, one that effectively balances de jure norms with de facto realities and centers Indigenous participation in environmental decision-making, and advocates for the establishment of binding regional agreements that move beyond non-binding declarations.

Taken together, these perspectives make a strong case for establishing an “Arctic Fossil Fuel Non-Proliferation Zone’‘ centered on climate justice and Indigenous leadership on the basis of what already recommended by different institutions, such as the WWF Arctic Programme [31] and similarly to what already proposed for the Amazon Region by the FF-NPT Initiative [87].

## 5 Conclusion

This study presents the first comprehensive Atlas of oil and gas production in the Arctic region based on openly accessible data. The findings revealed that approximately 512,306 km^2^ of the CAFF region are covered by concessions, representing 1.82% of the study area, a territory size comparable to Spain. Spatial analysis also identified a total of 44,539 onshore and offshore wells, 39,535 km of pipelines and 1,959,507 km of seismic lines across the Arctic region. The overlapping and proximity geographies suggest the presence of areas of overlapping interests or real conflict, where national resource interests intersect with local and Indigenous land use and ecological priorities. In these areas, proactive mediation and equitable land-use planning is needed to mitigate the potential or existing socio-ecological impacts of oil and gas activities. The results presented in this study corroborate previous works by Hanaček et al. [88] and Kröger [27], which identified the Arctic region as one of the regions at the frontier of oil & gas extractivism. While a just transition away from fossil fuels must avoid the disruption of the economies of producing countries, recent studies have shown that the development of new oil and gas reserves in the Arctic is no longer consistent with climate targets [3]. Furthermore, although the retreat of summer ice opens new opportunities for the expansion of the oil and gas frontier, current extraction and potential development are increasingly questioned due to the socio-ecological instability of the rapidly warming Arctic and the potential of reaching climate tipping points [24,27]. An example of this is the uncertainty regarding the deterioration and potential collapse of parts of Arctic oil and gas infrastructure due to permafrost thaw-related ground instability, as highlighted by Hjort et al. [89]. In light of these factors, the present study makes the case for an accelerated phase-out of Arctic oil and gas activities to mitigate both global climate disruption and the local socio-environmental harms of extraction. Hydrocarbon resources with the highest environmental impacts, such as those in the Arctic region and tropical rainforests, must be declared unburnable. What is needed, therefore, is a shift to a new paradigm that goes beyond techno economic criteria and that prioritises the preservation of what stays aboveground over the exploitation of what lies underground; a transformation of Arctic governance from a regime focused on access and extraction into one rooted in equity, ecology, and inclusion.

## Supporting information

S1 File Contains S1-S6 Tables and S1-S2 Appendices. (DOCX)
