# Supplementary material for: Unburnable carbon in the rapidly warming Arctic: Mapping spatial relationships among oil and gas development, ecologically sensitive areas and Indigenous Peoples’ lands
Source: PLoS One. 2026 Apr 22;21(4):e0345775. doi: 10.1371/journal.pone.0345775 (PMC13102241; doi:10.1371/journal.pone.0345775)
Supplement: S1 File — (DOCX) [file pone.0345775.s001.docx]

**Supporting information**

**S1 Table. Sources and details of regional oil and gas spatial information. For Canada, only provinces located above the CAFF boundary are included.**

**ALASKA**

| **Category** | **Data description** | **Details** | **Data format** | **Source** | **Acquisition Date** | **URL** |
| --- | --- | --- | --- | --- | --- | --- |
| **CONCESSIONS** | Oil Gas Lease Current | A dataset containing currently issued state of Alaska oil and gas leases.  <https://dog-soa-dnr.opendata.arcgis.com/datasets/SOA-DNR::oil-gas-lease-current/about> | shapefile | Alaska Department of Natural Resources - Division of Oil and Gas | 2024 | <https://dog-soa-dnr.opendata.arcgis.com/datasets/0db15f41eb624662952aee6c91787ac4_0/explore?location=67.600055%2C-147.672558%2C4.99> |
|  | BLM AK Oil and Gas Leases | This layer depicts authorized oil and gas leases with the National Petroleum Reserve - Alaska (NPRA) and Cook Inlet.  The authorized oil and gas leases dataset is produced on a regular basis by the Bureau of Land Management, Alaska State Office. <https://www.arcgis.com/sharing/rest/content/items/7485fd02528f412e9e1b6ceabe662aec/info/metadata/metadata.xml?format=default&output=html> | shapefile | Bureau of Land Management (BLM) – Alaska Cadastral Data | 2025 | https://gbp-blm-egis.hub.arcgis.com/maps  /7485fd02528f412e9e1b6ceabe662aec/about |
| **WELLS** | Well Surface Hole Location | No description | shapefile | Alaska Department of Natural Resources - Division of Oil and Gas | 2024 | <https://dog-soa-dnr.opendata.arcgis.com/datasets/923e69760d744410933a3df3912ece4b_0/explore> |
| **PIPELINES** | North Slope Infrastructure (V13): Roads, Pipelines and Developed Areas | Update 03/22/2023 | shapefile | Alaska Center for Conservation Science (ACCS) | 2024 | <https://catalog.northslopescience.org/dataset/2663> |
| **AREAS UNDER BID** | Lease Sale Tract Current | Dataset containing current lease sale tracts within the areawide boundary available for a lease sale. | shapefile | Alaska Department of Natural Resources - Division of Oil and Gas | 2024 | <https://dog-soa-dnr.opendata.arcgis.com/datasets/1923d5e123cc47feae362393aff10d13_0/explore?location=67.238096%2C-153.306202%2C5.34> |
| **SEISMIC LINES** | National Archive of Marine Seismic Surveys (NAMSS: A USGS data website of marine seismic reflection data within the U.S. Exclusive Economic Zone (EEZ) | The National Archive of Marine Seismic Surveys (NAMSS) is a marine seismic reflection profile data archive consisting of data acquired by or contributed to U.S. Department of the Interior agencies. The archived data were collected from 1975 to the present. They include marine seismic reflection profile data acquired originally for purposes of oil and gas exploration within the U.S. Exclusive Economic Zone (EEZ), a national scientific resource of inestimable value. The USGS is committed to safekeeping both agency and industry data on behalf of the academic community and the nation. These data are provided with free and open access. | shapefile | U.S. Geological Survey - U.S. Department of the Interior | 2024 | <https://www.usgs.gov/data/national-archive-marine-seismic-surveys-namss-a-usgs-data-website-marine-seismic-reflection>  <https://walrus.wr.usgs.gov/namss/> |
| **GAS FLARES** | Global Gas Flaring Observed from Space | Global Gas Flaring Observed from Space (filtered for Alaska) | Spreadsheet | Earth Observation Group | 2024 | <https://eogdata.mines.edu/products/vnf/global_gas_flare.html> |

**CANADA**

| Category | Sub-region | Data description | Details | Data format | Source | Acquisition Date | URL |
| --- | --- | --- | --- | --- | --- | --- | --- |
| **CONCESSIONS** | National - Northern Canada | Oil and gas rights | Last update: 2021-04-08  The Oil and Gas Rights dataset is Crown-Indigenous Relations and Northern Affairs Canada (CIRNAC) and Indigenous Services Canada (ISC) primary source for northern petroleum titles geographic location on maps. | shapefile | Government of Canada - Crown-Indigenous Relations and Northern Affairs Canada | 2024 | <https://open.canada.ca/data/en/dataset/208ddd6d-dea8-4d1c-bf62-5b49e8983a51>  <https://www.rcaanc-cirnac.gc.ca/eng/1100100036125/1583410876418> |
|  | Alberta | AER Order System Shapefile → Field Region | No description | shapefile | Alberta Energy Regulator | 2024 | <https://www.aer.ca/data-and-performance-reports/activity-and-data/spatial-data> |
|  | British Columbia | Oil and Gas Fields | A pool, or group of pools, within a specified geographic area that contain oil or natural gas. | shapefile | British Columbia Energy Regulator | 2024 | <https://data-bc-er.opendata.arcgis.com/datasets/6757a215a11a456ab1331ddaa4b9bfd1_0/explore?location=56.994200%2C-121.814802%2C5.83> |
|  | Ontario | NO CAFF | NO CAFF | NO CAFF | NO CAFF | NO CAFF | NO CAFF |
|  | Quebec | NO CAFF | NO CAFF | NO CAFF | NO CAFF | NO CAFF | NO CAFF |
|  | Newfoundland & Labrador | Oil and gas leases, Newfoundland & Labrador | Active licenses:  i) Active Exploration Licences  ii) Active Production Licences  iii) Active Significant Discovery Licences | shapefile | Canada-Newfoundland and Labrador Offshore Petroleum Board | 2024 | <https://home-cnlopb.hub.arcgis.com/pages/shapefiles> |
|  | Yukon | Oil and Gas Dispositions - 50k | Last update: 2020-12-09  Metadata: https://yukon.maps.arcgis.com/sharing/rest/content/items/af2104d0ff884b9c9929e9efc0286178/info/metadata/metadata.xml?format=default&output=html | shapefile | GeoYukon, the Government of Yukon's digital map data | 2024 | <https://mapservices.gov.yk.ca/geoyukon/?&LayerTheme=Oil%20and%20Gas> |
|  | Northwest Territories | Oil and Gas Rights | Northern Oil and Gas disposition and Call maps are available across Northern Canada and various regions within the jurisdiction of Crown-Indigenous Relations and Northern Affairs Canada. | .gdb, shapefile | Mineral and Petroleum Resources Division, Government of Northwest Territories  Government of Canada - Crown-Indigenous Relations and Northern Affairs Canada | 2024 | <https://www.iti.gov.nt.ca/en/services/oil-and-gas-rights-management/nwt-oil-and-gas-rights>  <https://www.rcaanc-cirnac.gc.ca/eng/1100100036125/1583410876418> |
|  | Nunavut | Oil and Gas Rights | Northern Oil and Gas disposition and Call maps are available across Northern Canada and various regions within the jurisdiction of Crown-Indigenous Relations and Northern Affairs Canada. | .gdb, shapefile | Mineral and Petroleum Resources Division, Government of Northwest Territories  Government of Canada - Crown-Indigenous Relations and Northern Affairs Canada | 2024 | <https://www.iti.gov.nt.ca/en/services/oil-and-gas-rights-management/nwt-oil-and-gas-rights>  <https://www.rcaanc-cirnac.gc.ca/eng/1100100036125/1583410876418> |
|  | Manitoba | NO CAFF | NO CAFF | NO CAFF | NO CAFF | NO CAFF | <https://www.gov.mb.ca/iem/petroleum/gis/index.html> |
|  | Saskatchewan | NO CAFF | NO CAFF | NO CAFF | NO CAFF | NO CAFF | <https://geohub.saskatchewan.ca/datasets/saskatchewan::oil-and-gas-pools-/explore?location=51.532988%2C-105.989543%2C6.61> |
| **WELLS** | National | Petroleum wells | No description  Data last updated: May 19, 2023 | shapefile | Government of Canada | 2024 | <https://open.canada.ca/data/en/dataset/8ba2aa2a-7bb9-4448-b4d7-f164409fe056>  →<https://ftp.maps.canada.ca/pub/nrcan_rncan/vector/canvec/shp/Res_MGT/> |
|  | National | Oil and Gas Wells in Canadian Basins | Oil and Gas Wells in Canadian Basins | Spreadsheet | Government of Canada - The BASIN Database | 2024 | https://basin.marine-geo.canada.ca/wells/index_e.php |
|  | Alberta | Surface Holes Shapefile | This dataset contains all Surface hole oil and gas wells in the Province of Alberta that have been applied for to the AER. This dataset includes information that is based on the ST37 report | shapefile | Alberta Energy Regulator | 2024 | <https://www.aer.ca/data-and-performance-reports/statistical-reports/st37> |
|  | British Columbia | Surface Hole | Provides non-confidential information about the surface location of a well. The status view provides information on the current status of the surface hole. | shapefile | British Columbia Energy Regulator | 2024 | <https://data-bc-er.opendata.arcgis.com/search?tags=OD_Well_Facility>  <https://www.bc-er.ca/files/gis/BCER-Open-Data-Licence.pdf> |
|  | Ontario | Petroleum Well | The locations and types of petroleum wells in the province.  The locations of wells that have been drilled for oil production, gas or salt resources or for underground storage of hydrocarbons.  This data can be used for land use and resource management, emergency management, as well as compliance and enforcement in the petroleum industry. The Data is collected on an on-going basis and maintained in the Ontario Petroleum Data System (OPDS).  Additional Documentation  Petroleum Well - Data Description  Petroleum Well - Documentation  Petroleum Well- User Guide  Status  On going: data is being continually updated  Maintenance and Update Frequency  Semi-monthly: data is updated twice a month  Contact  Petroleum Operations Section, Ministry of Natural Resources and Forestry, POSrecords@ontario.ca | shapefile | Petroleum Operations Section, Ministry of Natural Resources and Forestry - Land Information Ontario - Ontario GeoHub | May 2025 | <https://geohub.lio.gov.on.ca/datasets/lio::petroleum-well/about> |
|  | Quebec | Petroleum wells | Data last updated: May 19, 2023 | shapefile | Government of Canada | 2024 | <https://open.canada.ca/data/en/dataset/8ba2aa2a-7bb9-4448-b4d7-f164409fe056>  →https://ftp.maps.canada.ca/pub/nrcan_rncan/vector/canvec/shp/Res_MGT/ |
|  | Newfoundland & Labrador | Delineation Wells Shapefile  Development Wells Shapefile  Dual Classified Wells Shapefile  Exploration Wells Shapefile | This page provides access to all records available for disclosure in relation to each well, pursuant to exemptions outlined in subsections 119(5) and 119(9) of the Canada-Newfoundland and Labrador Atlantic Accord Implementation Act and subsections 115(5) and 115(9) of the Canada-Newfoundland and Labrador Atlantic Accord Implementation Newfoundland and Labrador Act. | shapefile | Canada Newfoundland & Labrador - Offshore Energy Regulator | 2025 | <https://open.canada.ca/data/en/dataset/8ba2aa2a-7bb9-4448-b4d7-f164409fe056>  <https://home-cnlopb.hub.arcgis.com/pages/well-inventory> |
|  | Yukon | Oil and Gas Wells - 50k | Last update: 2020-12-09  Metadata: <https://yukon.maps.arcgis.com/sharing/rest/content/items/11aabfb914c2469090d8488961b88e5d/info/metadata/metadata.xml?format=default&output=html> | shapefile | GeoYukon, the Government of Yukon's digital map data | 2025 | <https://mapservices.gov.yk.ca/geoyukon/?&LayerTheme=Oil%20and%20Gas> |
|  | Northwest Territories | Petroleum wells | No description  Data last updated: May 19, 2023 | shapefile | Government of Canada | 2024 | <https://open.canada.ca/data/en/dataset/8ba2aa2a-7bb9-4448-b4d7-f164409fe056>  →<https://ftp.maps.canada.ca/pub/nrcan_rncan/vector/canvec/shp/Res_MGT/> |
|  | Nunavut | Petroleum wells | No description  Data last updated: May 19, 2023 | shapefile | Government of Canada | 2024 | <https://open.canada.ca/data/en/dataset/8ba2aa2a-7bb9-4448-b4d7-f164409fe056>  →<https://ftp.maps.canada.ca/pub/nrcan_rncan/vector/canvec/shp/Res_MGT/> |
|  | Manitoba | NO CAFF | NO CAFF | NO CAFF | NO CAFF | NO CAFF | <https://www.gov.mb.ca/iem/petroleum/gis/index.html> |
|  | Saskatchewan | NO CAFF | NO CAFF | NO CAFF | NO CAFF | NO CAFF | <https://open.canada.ca/data/en/dataset/8ba2aa2a-7bb9-4448-b4d7-f164409fe056>  →<https://ftp.maps.canada.ca/pub/nrcan_rncan/vector/canvec/shp/Res_MGT/> |
| **PIPELINES** | National | Pipelines | No description | shapefile | Government of Canada | 2024 | <https://open.canada.ca/data/en/dataset/8ba2aa2a-7bb9-4448-b4d7-f164409fe056>  →[Index of /pub/nrcan_rncan/vector/canvec/shp/Res_MGT](https://ftp.maps.canada.ca/pub/nrcan_rncan/vector/canvec/shp/Res_MGT/) |
|  | Alberta | Pipelines | <https://static.aer.ca/prd/data/pipeline/EnhancedPipeline_Layout.pdf> | shapefile | Alberta Energy Regulator | 2024 | <https://www.aer.ca/data-and-performance-reports/activity-and-data/spatial-data> |
|  | British Columbia | Pipeline Segments (Permitted) | Pipeline centre-lines associated with oil and gas pipeline activity and falling within the area representing the pipeline right of way. | shapefile | British Columbia Energy Regulator | 2024 | <https://data-bc-er.opendata.arcgis.com/search?tags=OD_Pipeline> |
|  | Ontario | NO CAFF | NO CAFF | shapefile | Government of Canada | NO CAFF | <https://open.canada.ca/data/en/dataset/8ba2aa2a-7bb9-4448-b4d7-f164409fe056>  →[Index of /pub/nrcan_rncan/vector/canvec/shp/Res_MGT](https://ftp.maps.canada.ca/pub/nrcan_rncan/vector/canvec/shp/Res_MGT/) |
|  | Quebec | NO CAFF | NO CAFF | NO CAFF | NO CAFF | NO CAFF | https://open.canada.ca/data/en/dataset/8ba2aa2a-7bb9-4448-b4d7-f164409fe056  →[Index of /pub/nrcan_rncan/vector/canvec/shp/Res_MGT](https://ftp.maps.canada.ca/pub/nrcan_rncan/vector/canvec/shp/Res_MGT/) |
|  | Newfoundland and Labrador | Pipelines | No description | shapefile | Government of Canada | 2024 | https://open.canada.ca/data/en/dataset/8ba2aa2a-7bb9-4448-b4d7-f164409fe056  →<https://ftp.maps.canada.ca/pub/nrcan_rncan/vector/canvec/shp/Res_MGT/> |
|  | Yukon | NO CAFF | NO CAFF | NO CAFF | NO CAFF | NO CAFF | NO CAFF |
|  | Northwest Territories | Pipelines | No description | shapefile | Government of Canada | 2024 | https://open.canada.ca/data/en/dataset/8ba2aa2a-7bb9-4448-b4d7-f164409fe056  →https://ftp.maps.canada.ca/pub/nrcan_rncan/vector/canvec/shp/Res_MGT/ |
|  | Nunavut | Pipelines | No description | shapefile | Government of Canada | 2024 | https://open.canada.ca/data/en/dataset/8ba2aa2a-7bb9-4448-b4d7-f164409fe056  →https://ftp.maps.canada.ca/pub/nrcan_rncan/vector/canvec/shp/Res_MGT/ |
|  | Manitoba | NO CAFF | NO CAFF | NO CAFF | NO CAFF | NO CAFF | NO CAFF |
|  | Saskatchewan | NO CAFF | NO CAFF | NO CAFF | NO CAFF | NO CAFF | <https://geohub.saskatchewan.ca/datasets/saskatchewan::pipelines-and-flowlines/explore?layer=0&location=53.159681%2C-94.225937%2C4.52> |
| **AREAS UNDER BID** | Alberta | NO CAFF | NO CAFF | NO CAFF | NO CAFF | NO CAFF | NO CAFF |
|  | British Columbia | NO CAFF | NO CAFF | NO CAFF | NO CAFF | NO CAFF | NO CAFF |
|  | Ontario | NO CAFF | NO CAFF | NO CAFF | NO CAFF | NO CAFF | NO CAFF |
|  | Quebec | NO CAFF | NO CAFF | NO CAFF | NO CAFF | NO CAFF | NO CAFF |
|  | Newfoundland & Labrador | CALL FOR BIDS (2014-Present) | No description | shapefile | Canada Newfoundland & Labrador - Offshore Energy Regulator | 2024 | <https://home-cnlopb.hub.arcgis.com/pages/land-tenure#CFB> |
|  | Yukon | Oil and Gas Bid Locations - 50k | Last update: 2020-12-09  Metadata: https://yukon.maps.arcgis.com/sharing/rest/content/items/bc5a6c35e2dd444e961e9d656fada280/info/metadata/metadata.xml?format=default&output=html | shapefile | GeoYukon, the Government of Yukon's digital map data | 2025 | <https://open.yukon.ca/data/oil-and-gas-bid-locations-50k>  <https://mapservices.gov.yk.ca/geoyukon/?&LayerTheme=Oil%20and%20Gas> |
|  | Northwest Territories | Call for Bids | Call Maps and Shapefile Downloads  Maps and Shapefiles for current and past calls are available for download. Tip: use the "Filter items" to quickly find specific Call regions, years, Shapefiles, etc. | shapefile | Government of Canada - Crown-Indigenous Relations and Northern Affairs Canada | 2024 | <https://www.rcaanc-cirnac.gc.ca/eng/1100100036125/1583410876418> |
|  | Nunavut | Call for Bids | Call Maps and Shapefile Downloads  Maps and Shapefiles for current and past calls are available for download. Tip: use the "Filter items" to quickly find specific Call regions, years, Shapefiles, etc. | shapefile | Government of Canada - Crown-Indigenous Relations and Northern Affairs Canada | 2024 | <https://www.rcaanc-cirnac.gc.ca/eng/1100100036125/1583410876418> |
|  | Manitoba | NO CAFF | NO CAFF | NO CAFF | NO CAFF | NO CAFF | NO CAFF |
|  | Saskatchewan | NO CAFF | NO CAFF | NO CAFF | NO CAFF | NO CAFF | NO CAFF |
| **SEISMIC LINES** | National | Geophysical Surveys | Geophysical Surveys in Canadian Basins | spreadsheet | Government of Canada - The BASIN Database | 2024 | https://basin.marine-geo.canada.ca/seismic/index_e.php |
|  | Alberta | Wall-to-Wall Human Footprint Inventory - Year 2022  HFI2022_v1_1 — o20_SeismicLines_and_Trails_CenterLines_HFI_2022 | A comprehensive digital representation of anthropogenic disturbances (e.g., agriculture, forestry, energy) on the Alberta land-base, digitized manually, as seen from SPOT6 satellite imagery. This includes linear features. | .gdb | Alberta Biodiversity Monitoring Institute | 2025 | <https://abmi.ca/data-portal/80.html> |
|  | British Columbia | Geophysical Lines (Permitted) [BCOGC-44932] | Geophysical exploration is an energy resource activity under the Energy Resource Activities Act (ERAA) and is specifically defined in the Petroleum and Natural Gas Act (PNG) Act as investigation of the subsurface by seismic, gravimetric, magnetic, electric and geochemical operations and by any other method approved by the BC Energy Regulator, but does not include the use of geophysical well logs, vertical seismic profile surveys or other surveys obtained from a well. This dataset contains line features for approved and final plan geophysical activity collected on or after Oct. 30, 2006. | shapefile | British Columbia Energy Regulator | 2024 | <https://www.bc-er.ca/data-reports/data-centre/?category=44925> |
|  | Ontario | Seismic Reflection | No description | shapefile | Government of Canada | 2024 | <http://ftp.maps.canada.ca/pub/nrcan_rncan/raster/marine_geoscience/Seismic_Reflection_Scanned/> |
|  | Quebec | Seismic Reflection | No description | shapefile | Government of Canada | 2024 | <http://ftp.maps.canada.ca/pub/nrcan_rncan/raster/marine_geoscience/Seismic_Reflection_Scanned/> |
|  | Newfoundland & Labrador | Seismic Reflection | No description | shapefile | Government of Canada | 2024 | <https://home-cnlopb.hub.arcgis.com/pages/geophysical> |
|  | Yukon | Seismic Reflection | No description | shapefile | GeoYukon, the Government of Yukon's digital map data | 2024 | <http://ftp.maps.canada.ca/pub/nrcan_rncan/raster/marine_geoscience/Seismic_Reflection_Scanned/>  <https://open.yukon.ca/data/oil-and-gas-seismic-lines/resource/f2d47e9f-c966-3e86-b268-8334334106f0> |
|  | Northwest Territories | Seismic Reflection | No description | shapefile | Government of Canada | 2024 | <http://ftp.maps.canada.ca/pub/nrcan_rncan/raster/marine_geoscience/Seismic_Reflection_Scanned/> |
|  | Nunavut | Seismic Reflection | No description | shapefile | Government of Canada | 2024 | <http://ftp.maps.canada.ca/pub/nrcan_rncan/raster/marine_geoscience/Seismic_Reflection_Scanned/> |
|  | Manitoba | Seismic Reflection | No description | shapefile | Government of Canada | 2024 | <http://ftp.maps.canada.ca/pub/nrcan_rncan/raster/marine_geoscience/Seismic_Reflection_Scanned/> |
|  | Saskatchewan | NO CAFF | NO CAFF | NO CAFF | NO CAFF | NO CAFF | <http://ftp.maps.canada.ca/pub/nrcan_rncan/raster/marine_geoscience/Seismic_Reflection_Scanned/> |
| **GAS FLARES** | National | Global Gas Flaring Observed from Space | Filtered for Canada | Spreadsheet | Earth Observation Group | 2024 | <https://eogdata.mines.edu/products/vnf/global_gas_flare.html> |

**GREENLAND**

| Category | Data description | Details | Data format | Source | Acquisition Date | URL |
| --- | --- | --- | --- | --- | --- | --- |
| **CONCESSIONS** | Greenland hydrocarbon licenses | Oil exploration license | shapefile | Greenland Bureau of Minerals and Petroleum (BMP)  Now: Mineral Licence and Safety Authority, Greenland | 2024 | <https://portal.govmin.gl/map> |
| **WELLS** | Exploration wells in Greenland | Drilled hydrocarbon exploration wells | shapefile | GEUS, Danish Geological Survey | 2024 | <http://www.bmp.gl/petroleum/exploration-wells>  <https://data.geus.dk/geusmap/?mapname=subsurface_grl#baslay=baseMapGl&optlay=&extent=-1655133.8895722213,7142433.392057314,1662726.1927323057,8600477.373538796&layers=northpole_graticule,samba_wells_grl&filter_1=well_name%3D%26label.config%3D> |
| **PIPELINES** | Not present | Not present | Not present | Not present | Not present | Not present |
| **AREAS UNDER BID** | Areas under bid | No description | shapefile | The Oil and Gas Department, Government of Greenland;  GEUS, Danish Geological Survey | 2021 | <https://naalakkersuisut.gl/en/Naalakkersuisut/News/2021/07/1507_oliestop>  **No longer available**  A representation of these areas is available at this link: https://polarjournal.net/what-does-greenlands-halt-of-oil-production-plans-mean/ |
| **SEISMIC LINES** | 2D lines | No description | shapefile | GEUS, Danish Geological Survey | 2024 | <https://data.geus.dk/geusmap/?mapname=subsurface_grl#baslay=baseMapGl&optlay=&extent=-839957.1256254539,7491131.771398876,2477902.956679073,8949175.752880357&layers=northpole_graticule,seismic_lines_grl&filter_1=line_name%3D%26survey_name%3D%26survey_type.part%3D%26recording_period%3D> |
| **GAS FLARES** | Global Gas Flaring Observed from Space | Filtered for Greenland | Spreadsheet | Earth Observation Group | 2024 | <https://eogdata.mines.edu/products/vnf/global_gas_flare.html> |

**NORWAY**

| Category | Data description | Details | Data format | Source | Acquisition Date | URL |
| --- | --- | --- | --- | --- | --- | --- |
| **CONCESSIONS** | Licence | Current production licence areas, “active” with overall geometry, “inactive” without geometry. | shapefile | Norwegian Offshore Directorate | 2025 | <https://www.sodir.no/en/facts/data-and-analyses/open-data/> |
| **WELLS** | Wellbore | Exploration, development and shallow wellbores. | shapefile | Norwegian Offshore Directorate | 2024 | https://www.sodir.no/en/facts/data-and-analyses/open-data/ |
| **PIPELINES** | TUF | Main pipelines. The dataset contains not infield pipelines. | shapefile | Norwegian Offshore Directorate | 2024 | <https://www.sodir.no/en/facts/data-and-analyses/open-data/> |
| **AREAS UNDER BID** | Licencing APA | Updated APA (Awards in predefined areas) gross areas. | shapefile | Norwegian Offshore Directorate | 2025 | <https://www.sodir.no/en/facts/data-and-analyses/open-data/> |
| **SEISMIC LINES** | Survey | Areas with planned survey after 1.1. 2009. The dataset contains both gross (included turn area for the boat) and net (acquisition area) for seismic, electromagnetic, site and basement surveys.  Polygon data were filtered by acquisition area (‘NetGross’ attribute = ’Net’) and 2D seismic (‘surParTyp’ attribute = ‘2D’) and then collapsed to linear format. | shapefile | Norwegian Offshore Directorate | 2024 | <https://www.sodir.no/en/facts/data-and-analyses/open-data/> |
| **GAS FLARES** | Global Gas Flaring Observed from Space | Filtered for Norway | spreadsheet | Earth Observation Group | 2024 | <https://eogdata.mines.edu/products/vnf/global_gas_flare.html> |

**RUSSIA**

| Category | Data description | Details | Data format | Source | Acquisition Date | URL |
| --- | --- | --- | --- | --- | --- | --- |
| **CONCESSIONS** | Oil & Gas Fields | Defined as “areas above a hydrocarbon accumulation” | shapefile | Sabbatino et al., 2017 - Global Oil & Gas Features Database | 2024 | <https://edx.netl.doe.gov/dataset/global-oil-gas-features-database> |
| **WELLS** | Oil & Gas Wells | The wells dataset is not in the form of a points vector, but as a regular square grid of 5 km side, where each cell contain the estimated absolute number of wells | shapefile | Sabbatino et al., 2017 - Global Oil & Gas Features Database | 2024 | <https://edx.netl.doe.gov/dataset/global-oil-gas-features-database> |
| **PIPELINES** | Oil & Gas Pipelines | No description | shapefile | OpenStreetMap  Sabbatino et al., 2017 - Global Oil & Gas Features Database | 2022 | Open Street Map  Crossed with maps published in World Energy Atlas (2013) and company data that confirmed and validated OSM ones |
| **AREAS UNDER BID** | No data | No data | No data | No data | No data | No data |
| **SEISMIC LINES** | No data | No data | No data | No data | No data | No data |
| **GAS FLARES** | Global Gas Flaring Observed from Space | Filtered for Russia | spreadsheet | Earth Observation Group | 2024 | <https://eogdata.mines.edu/products/vnf/global_gas_flare.html> |

**OTHER LAYERS**

| Category | Details | Data format | Source | Acquisition Date | URL |
| --- | --- | --- | --- | --- | --- |
| Indigenous Peoples’ land | In Garnett et al (2018), IPLs refer to areas where Indigenous land tenure is formally recognized and where Indigenous communities, according to available data, maintain significant de facto influence over land management, defined as “the process of determining the use, development and care of land resources in a manner that fulfils material and non-material cultural needs, including livelihood activities such as hunting, fishing, gathering, resource harvesting, pastoralism and small scale agriculture and horticulture.” (Garnett et al., 2018, p. 370). | shapefile | Request to Garnett et al., 2018 | 2024 | <https://www.nature.com/articles/s41893-018-0100-6> |
| Protected Areas | https://www.protectedplanet.net/en/thematic-areas/wdpa?tab=Methodology | shapefile | UNEP-WCMC, IUCN | 2024 | https://www.protectedplanet.net/ |
| Key biodiversity areas | https://www.keybiodiversityareas.org/about-kbas/what-are-kbas | shapefile | KBA Partnership: BirdLife International, International Union for the Conservation of Nature, American Bird Conservancy, Amphibian Survival Alliance, Conservation International, Critical Ecosystem Partnership Fund, Global Environment Facility, Re:wild, NatureServe, Rainforest Trust, Royal Society for the Protection of Birds, Wildlife Conservation Society and World Wildlife Fund. | 2024 | Request to BirdLife: <http://keybiodiversityareas.org/kba-data/request> |
| Global 200 Ecoregions (WWF) | WWF's Global 200 is a first attempt to identify a set of ecoregions whose conservation would achieve the goal of saving a broad diversity of the Earth's ecosystems. These ecoregions include those with exceptional levels of biodiversity, such as high species richness or endemism, or those with unusual ecological or evolutionary phenomena. Tropical rain forests deservedly garner much conservation attention, as they may contain half of the world's species. However, a comprehensive strategy for conserving global biodiversity must strive to include the other 50 percent of species and habitats, such that all species and the distinctive ecosystems that support them are conserved. Habitats like tropical dry forests, tundra, polar seas, desert springs and mangroves all harbor unique species, communities, adaptations and phenomena. To lose examples of these assemblages would represent an enormous loss of global biodiversity. For this reason, the Global 200 aims to represent all of the world's biodiversity by identifying outstanding ecoregions in all of the world's biomes and biogeographic realms. The Global 200 ecoregions represent those ecoregions where WWF is initially focusing its ecoregion conservation efforts to develop biodiversity visions. Ecoregion conservation is currently well underway in many of WWF's Global 200 ecoregions. Learn more about this work in WWF documents and reports from priority ecoregions. | shapefile | Olson, D.M., E. Dinerstein, E.D. Wikramanayake, N.D. Burgess, G.V.N. Powell, E.C. Underwood, J.A. D'Amico, H.E. Strand, J.C. Morrison, C.J. Loucks, T.F. Allnutt, J.F. Lamoreux, T.H. Ricketts, I. Itoua, W.W. Wettengel, Y. Kura, P. Hedao, and K. Kassem. 2001. Terrestrial ecoregions of the world: A new map of life on Earth. BioScience 51(11):933-938. | 2024 | [World Wildlife Fund - Global 200 (terrestrial) Ecoregions \| Data Basin](https://databasin.org/datasets/a5b34649cc69417ba52ac8e2dce34c3b/) |
| Last of the Wild areas | Last of the Wild, v2, represents the least human influenced or wild areas of major terrestrial biomes based on the Human Footprint Index data set and Topographic basemap. | shapefile | Venter, O., Sanderson, E. W., Magrach, A., Allan, J. R., Beher, J., Jones, K. R., Levy, M. A., & Watson, J. E. (2018). Last of the Wild Project, Version 3 (LWP-3): 2009 Human Footprint, 2018 Release (Version 2018.00) [Data set]. Palisades, NY: NASA Socioeconomic Data and Applications Center (SEDAC). https://doi.org/10.7927/H46T0JQ4 Date Accessed: 2025-12-22 | 2024 | <https://www.earthdata.nasa.gov/data/catalog/sedac-ciesin-sedac-lwp3-hf-2009-2018.00> |
| Geographic range Rangifer tarandus | https://www.iucnredlist.org/species/29742/22167140 | shapefile | IUCN - Red List | 2024 | Request to IUCN [Rangifer tarandus (Reindeer) (iucnredlist.org)](https://www.iucnredlist.org/species/29742/22167140) |
| Geographic range Gavia adamsii | https://www.iucnredlist.org/species/22697847/132607949 | shapefile | IUCN - Red List | 2024 | Request to IUCN [Gavia adamsii (Yellow-billed Loon) (iucnredlist.org)](https://www.iucnredlist.org/species/22697847/132607949) |
| Geographic range Ursus maritimus | https://www.iucnredlist.org/species/22823/14871490 | shapefile | IUCN - Red List | 2024 | Request to IUCN [Ursus maritimus (Polar Bear) (iucnredlist.org)](https://www.iucnredlist.org/species/22823/14871490) |
| CAFF boundaries | Boundaries of the geographic area covered by the Arctic Biodiversity Assessment. Includes sub, low and high Arctic boundaries | shapefile | CAFF - Arctic Biodiversity Data Service (ABDS) | 2024 | <https://geo.abds.is/geonetwork/srv/eng/catalog.search#/metadata/f0eb86a7-e408-4138-9432-dedb991f13d1> |
| Marine and land zones | This dataset combines the boundaries of the world countries and the Exclusive Economic Zones of the world. It was created by combining the ESRI world country database and the EEZ V11 dataset. | shapefile | Flanders Marine Institute (2020). Union of the ESRI Country shapefile and the Exclusive Economic Zones (version 3). | 2024 | <https://www.marineregions.org/> →<https://doi.org/10.14284/403> (created from EEZ version 11) |
| EEZ | Maritime Boundaries and Exclusive Economic Zones from the VLIZ Maritime Boundaries Geodatabase. Boundaries have been built using information about treaties between coastal countries. When treaties are not available, median lines have been calculated. An exclusive economic zone (EEZ) is a seazone extending from a state's coast or baseline over which the state has special rights over the exploration and use of marine resources. Generally a state's EEZ extends 200 nautical miles out from its coast, except where resulting points would be closer to another country. This dataset also contains delimitation of overlapping claims and joint regimes. more  In the Maritime Boundaries Geodatabase, Marine Regions makes available most of the maritime areas defined in the Law of the Sea Convention: Exclusive Economic Zones (EEZ), Territorial Seas (TS), Contiguous Zones (CZ), Internal Waters (IW) and Archipelagic Waters (AW). | shapefile | Flanders Marine Institute (2019). Maritime Boundaries Geodatabase: Maritime Boundaries and Exclusive Economic Zones (200NM), version 11. | 2024 | <https://www.marineregions.org/>.  →<https://doi.org/10.14284/386> |

**S1 Appendix. NGO conservation priority layers: selection criteria and spatial overlap in the CAFF Region**

NGO-led conservation projects were selected based on the layers included in the conservation priority index developed by the widely used Co$tingNature model [1], which integrates multiple globally recognized datasets. These include Endemic Bird Areas (BirdLife International), Global 200 Ecoregions (WWF), Biodiversity Hotspots (Conservation International), Last of the Wild (WCS and CIESIN), Important Bird Areas (BirdLife International), and Key Biodiversity Areas (coordinated by IUCN, in collaboration with BirdLife International, Plantlife International, and Conservation International). Of these six layers, only four spatially overlap with the CAFF region (namely Global 200 Ecoregions, LW, IBAs, and KBAs). However, within the CAFF boundary, the IBAs dataset considered for this study were found to spatially coincide with the KBAs dataset. This correspondence reflects the fact that the criteria underpinning IBA identification were adapted to create KBAs, therefore many IBAs have been formally adopted as KBAs through the KBA Partnership process [2]. Consequently, in the CAFF region considered here, IBAs do not provide additional spatial information beyond that captured by the KBAs layer. For this reason, and to avoid redundancy, this study refers exclusively to KBAs.

References:

1. Mulligan M. Documentation for the Co$tingNature Model V3 [Internet]. Feb 9, 2024 update [cited 2025 Dec 22]. Original V3 documentation 2017. Available from: <https://docs.google.com/document/d/136OvAO6PSyVBp0gNl9f0_pIAIg-h-4JZGArnf2V6-Us/edit?tab=t.0>
2. BirdLife International. The criteria used to identify IBAs have evolved into a global standard [Internet]. 2023 [cited 2025 Dec 23]. Available from: https://datazone.birdlife.org/articles/the-criteria-used-to-identify-ibas-have-evolved-into-a-global-standard

**S2 Table. Spatial relationships between oil and gas elements and PAs**

| **PROTECTED AREAS** | **Norway** | **Greenland** | **Alaska** | **Russia** | **Canada** | **Arctic** |
| --- | --- | --- | --- | --- | --- | --- |
| PAs in concessions (km^2^) (% in concession) | 388 (1.55%) | 3,194 (17.39%) | 15 (0.07%) | 34,554 (9.75%) | 637 (0.68%) | 38,787 (7.57%) |
| PAs in bidding areas (km^2^) (% in bidding area) | 0 (0%) | 899 (0.11%) | 1,542 (2.72%) | 0 (0%) | 0 (0%) | 2,441 (0.22%) |
| Wells on PAs (n) (% on total wells) | 8 (1.37%) | 44 (33.08%) | 12 (0.15%) | 125 (4.57%) | 214 (0.65%) | 403 (0.90%) |
| Pipeline on PAs (km) (% on total pipelines) | 0 (0%) | 0 (0%) | 0 (0%) | 73 (1.09%) | 309 (0.98%) | 382 (0.97%) |

**S3 Table. Spatial relationships between oil and gas elements and species ranges**

| **SPECIES RANGES** | Norway | Greenland | Alaska | Russia | Canada | ARCTIC |
| --- | --- | --- | --- | --- | --- | --- |
| **SPECIES RANGES IN CONCESSIONS (% on concessions)** | | | | | | |
| *Gavia adamsii* | 4,691 (18,87%) | 0 (0%) | 18,682 (87.45%) | 193,485 (54.62%) | 285 (0.30%) | 217,143 (42.38%) |
| *Rangifer tarandus* | 0 (0%) | 0 (0%) | 18,630 (87.21%) | 68,779 (19.42%) | 68,447 (73.22%) | 155,857 (30.42%) |
| *Ursus maritimus* | 950 (3.82%) | 18,367 (100%) | 21,363 (100%) | 191,692 (54.11%) | 26,657 (28.52%) | 259,029 (50.56%) |
| Number of ranges overlapping the oil and gas concessions | 2 | 1 | 3 | 3 | 3 |  |
| One species | 5,642(22.69%) | 18,367 (100%) | 2,680 (12.55%) | 183,263 (51.73%) | 91,833 (98.24%) | 301,785 (58.91%) |
| Two species overlapping | 0 (0%) | 0 (0%) | 52 (0.25%) | 61,227 (17.28%) | 1377 (1.47%) | 62,657 (12.23%) |
| Three species overlapping | 0 (0%) | 0 (0%) | 18630 (87.21%) | 49,413 (13.95%) | 267 (0.29%) | 68,310 (13.33%) |
| **SPECIES RANGES IN BIDDING AREAS (% bidding areas)** | | | | | | |
| *Gavia adamsii* | 28,651 (15.56%) | 0 (0%) | 30,963 (54.61%) | 0 (0%) | 0 (0%) | 59,614 (5.49%) |
| *Rangifer tarandus* | 0 (0%) | 4,527 (0.54%) | 45,996 (81.13%) | 0 (0%) | 0 (0%) | 50,523 (4.65%) |
| *Ursus maritimus* | 71,163 (38.64%) | 836,459 (99.67%) | 34,526 (60.90%) | 0 (0%) | 6,062 (100.00%) | 948,210 (87.30%) |
| **WELLS IN SPECIES RANGES (% total wells)** | | | | | | |
| One species | 350 (60.03%) | 117 (87.97%) | 723 (8.85%) | 1,598 (58.47%) | 32,726 (99.38%) | 35,514 (79.73%) |
| Two species overlapping | 0 (0%) | 16 (12.03%) | 25 (0.31%) | 324 (11.86%) | 170 (0.52%) | 535 (1.20%) |
| Three species overlapping | 0 (0%) | 0 (0%) | 7,400 (90.63%) | 276 (10.10%) | 25 (0.08%) | 7,701 (17.29%) |
| **PIPELINE IN SPECIES RANGES (% pipeline)** | | | | | | |
| One species | 143 (71.70%) | 0 (0%) | 137 (11.09%) | 3,665 (55.01%) | 31,273 (99.47%) | 35,218 (89.08%) |
| Two species overlapping | 0 (0%) | 0 (0%) | 32 (2.61%) | 1,328 (19.93%) | 157 (0.50%) | 1,517 (3.84%) |
| Three species overlapping | 0 (0%) | 0 (0%) | 1,065 (86.30%) | 544 (8.16%) | 9 (0.03%) | 1,618 (4.09%) |

**S4 Table. Spatial relationships between oil and gas elements and conservation priorities**

| **CONSERVATION PRIORITIES** | Norway | Greenland | Alaska | Russia | Canada | ARCTIC |
| --- | --- | --- | --- | --- | --- | --- |
| **CONSERVATION PRIORITIES IN CONCESSIONS (% concessions)** | | | | | | |
| KBA | 0 (0%) | 2,704 (14.72%) | 2,680 (12.55%) | 19,635 (5.54%) | 1,859 (1.99%) | 26,879 (5.25%) |
| LW | 0 (0%) | 8,250 (44.92%) | 15,192 (71.11%) | 287,274 (81.10%) | 56,379 (60.31%) | 367,095 (71.66%) |
| WWF ecoregions | 0 (0%) | 0 (0%) | 18,515 (86.67%) | 21,590 (6.09%) | 40,557 (43.39%) | 80,662 (15.74%) |
| One conservation priority | 0 (0%) | 5,680 (30.93%) | 5,541 (25.94%) | 260,219 (73.46%) | 27,066 (28.95%) | 298,507 (58.27%) |
| Two conservation priorities overlapping | 0 (0%) | 2,637 (14.36%) | 14,959 (70.02%) | 31,469 (8.88%) | 33,916 (36.28%) | 82,981 (16.20%) |
| Three conservation priorities overlapping | 0 (0%) | 0 (0%) | 309 (1.45%) | 1,780 (0.50%) | 1,299 (1.39%) | 3,389 (0.66%) |
| Number of conservation priorities overlapping oil and gas concessions | 0 | 2 | 3 | 3 | 3 |  |
| **CONSERVATION PRIORITIES IN BIDDING AREAS (% bidding areas)** | | | | | | |
| KBA | 0 (0%) | 747 (0.09%) | 13,098 (23.10%) | 0 (0%) | 0 (0%) | 13,845 (1.27%) |
| LW | 0 (0%) | 12,479 (1.49%) | 38,181 (67.34%) | 0 (0%) | 0 (0%) | 50,660 (4.66%) |
| WWF ecoregions | 0 (0%) | 0 (0%) | 26,135 (46.10%) | 0 (0%) | 0 (0%) | 26,135 (2.41%) |
| Number of conservation priorities overlapping the bidding areas | 0 | 2 | 3 | 0 | 0 |  |
| **WELLS IN CONSERVATION PRIORITIES (% on wells)** | | | | | | |
| One conservation priority | 5 (0.86%) | 57 (42.86%) | 6,518 (79.83%) | 2,488 (91.04%) | 15,549 (47.22%) | 24,617 (55.27%) |
| Two conservation priorities overlapping | 0 (0%) | 20 (15.04%) | 1,077 (13.20%) | 205 (7.50%) | 11,672 (35.44%) | 12,974 (29.13%) |
| Three conservation priorities overlapping | 0 (0%) | 0 (0%) | 60 (0.76%) | 2 (0.07%) | 76 (0.23%) | 138 (0.31%) |
| **PIPELINE IN CONSERVATION PRIORITIES (% pipeline)** | | | | | | |
| One conservation priority | 4 (1.79%) | 0 (0%) | 885 (71.76%) | 4,958 (74.42%) | 14,690 (46.72%) | 20,537 (51.95%) |
| Two conservation priorities overlapping | 0 (0%) | 0 (0%) | 123 (10.01%) | 440 (6.61%) | 9,976 (31.73%) | 10,540 (26.66%) |
| Three conservation priorities overlapping | 0 (0%) | 0 (0%) | 0 (0%) | 1 (0.01%) | 320 (1.02%) | 321 (0.81%) |

**S5 Table. Spatial relationships between oil and gas elements and IPLs**

| **INDIGENOUS PEOPLES’ LANDS** | **Norway** | **Greenland** | **Alaska** | **Russia** | **Canada** | **ARCTIC** |
| --- | --- | --- | --- | --- | --- | --- |
| **IPLs (CAFF) (**km^2^**)** | 92,542 | 2,150,885 | 100,801 | 4,325,359 | 559,228 | 7,228,816 |
| % on total lands | 58.48 | 99.17 | 16.38 | 80.06 | 10.39 | 52.67 |
| **IPLs in concessions (**km^2^**)** | 0 | 8,294 | 385 | 285,085 | 2,573 | 296,338 |
| % on IPL | 0 | 0.39 | 0.38 | 6.59 | 0.46 | 4.10 |
| % on concessions | 0 | 99.86 | 2.08 | 92.27 | 3.76 | 73.30 |
| **IPLs in Bidding areas (**km^2^**)** | 0 | 12,482 | 2,755 | 0 | 0 | 15,237 |
| % on IPLs | 0 | 0.58 | 2.73 | 0 | 0 | 0.21 |
| % on bidding area | 0 | 0 | 4,86 | 0 | 0 | 1,40 |
| **Pipelines on IPLs** | 0 | 0 | 34 | 5,516 | 384 | 5,934 |
| % on total pipelines for each country | 0% | 0% | 2.74% | 83.83% | 1.22% | 15.12% |
| **Wells on IPLs** | 0 | 86 | 429 | 2,428 | 203 | 3,146 |
| % on total wells for each country | 0% | 95.56% | 5.47% | 89.96% | 0.62% | 7.24% |

##

**S2 Appendix. Availability and status interpretation of wells, bidding and concessions datasets**

## Availability and completeness of well ‘spud date’ and ‘activity’ information across countries

### Motivation

### This analysis was conducted to clarify the interpretability of the methods, specifically (i) whether historical wells are included (e.g. wells that are no longer active), (ii) how the start of drilling activity, (iii) how missing temporal information should be interpreted. And (iv) whether third-party datasets were sufficiently complete to enable the analysis of temporal dimension of the overlap. By doing so, this analysis strengthens the methodological transparency and the assessment of the validity of the spatial intersections between oil and gas wells, ecologically sensitive areas, and Indigenous Peoples’ lands. To this end, we performed a systematic assessment of the temporal attributes and well status information available in each national and regional well dataset used in the analysis.

### Definition of spud date

In the oil and gas industry, a *spudded well* refers to a well for which drilling operations have officially begun. The *spud date* corresponds to the moment when the drill bit first penetrates the ground, marking the effective start of drilling activities. This date represents a key temporal attribute for characterizing well development and activity timing.

In this study, we assessed the availability, semantic consistency, and completeness of spud date information across multiple national and regional well datasets. Where a true ‘spud date’ was not available, we identified and documented the use of proxy temporal attributes (e.g. ‘administrative dates’ such as licence issuance or well authorization dates), explicitly distinguishing them from actual drilling start dates.

### Methodological criteria

For each dataset, the following aspects were evaluated:

- **Presence of a temporal attribute**Whether a field explicitly representing the spud date, or a documented proxy for the start of well activity, is available.
- **Semantic meaning of the attribute**Whether the attribute represents:
  - the actual start of drilling (*spud date*), or
  - an administrative or regulatory milestone (e.g. licence issued, authorization granted).
- **Completeness**
  - *Complete*: the attribute is populated for all wells in the dataset.
  - *Partial*: the attribute exists but contains NULL values.
  - *Absent*: no relevant temporal attribute is available.
- **Notes on NULL values and interpretation**When spud date values were missing, we investigated whether NULLs were systematically associated with specific well statuses (e.g. *Expired*, *Cancelled*, *Abandoned*). In such cases, missing values were interpreted as structurally meaningful rather than data errors

### Results

#### Table A – Availability and completeness of spud date (or ‘administrative date’) information by country/region

| Country / Region | Temporal attribute used (spud date or ‘administrative date’) | Temporal data available | Completeness | Notes on NULL values and interpretation |
| --- | --- | --- | --- | --- |
| Greenland | Spudded | Yes | Complete | Spud date available for all wells; consistent with well status |
| Norway | EntryDate (“date when the wellbore was spudded”) | Yes | Partial | 72 NULL values out of 583 wells. Of these, NULL wells 50 wells have purpose = NULL, suggesting non-drilled or reclassified wells  METADATA: <https://factpages.sodir.no/en/wellbore/attributes#:~:text=Norwegian%20Offshore%20Directorate%27s%20unique%20id%20for%20the%20field.&text=NPDID%20drilling%20facility-,Norwegian%20Offshore%20Directorate%27s%20unique%20id%20for,that%20has%20drilled%20the%20wellbore.&text=NPDID%20production%20facility-,Norwegian%20Offshore%20Directorate%27s%20unique%20id,production%20facility%2C%20for%20development%20wellbores.&text=wellbore%20reclassified%20from-,Norwegian%20Offshore%20Directorate%27s%20unique%20id%20for,this%20wellbore%20was%20reclassified%20from.&text=The%20NPDs%20unique%20ID%20for,from%20%28well%20head%20position%29.&text=The%20number%20of%20the%20day,the%20earth%27s%20crust%20/%20sea%20floor.&text=The%20number%20of%20month%20in,the%20earth%27s%20crust%20/%20sea%20floor.&text=For%20exploration%20wellbores:%20Floating%20and,casing%20or%20liner%20was%20set.&text=For%20exploration%20wellbores:%20Floating%20and,casing%20or%20liner%20was%20set> |
| Alaska (USA) | SpudDate | Yes | Partial | 560 NULL values out of 8,165 wells; NULLs mainly associated with permit expired, permit cancelled, or undefined status |
| Russia | – | No | Absent | No spud date or equivalent temporal attribute available |
| Canada – Government of Canada | Datemin and datemax | Yes (unclear) | Complete | Temporal attribute unclear |
| Canada – The BASIN Database | Original Spud Year | Yes | Complete | Spud date available for all wells, mostly P&A wells |
| Canada – Northwest Territories | Datemin and datemax | Yes (unclear) | Complete | Temporal attribute unclear |
| Canada – Nunavut | Datemin and datemax | Yes (unclear) | Complete | Temporal attribute unclear |
| Canada – Newfoundland & Labrador | SpuDate | Yes | Partial | 37 NULL values out of 122 wells |
| Canada – Alberta | LicStatDat (Licence Issued Date) | Yes (‘administrative date’) | Complete | Administrative date; does not necessarily correspond to drilling start |
| Canada – British Columbia | WELL_AUT_1 (Well Authorization Granted Date) | Yes (‘administrative date’) | Complete | Additional temporal fields available (WELL_NAME_, STATUS_EFF) |
| Canada – Yukon | ISSUED | Yes (‘administrative date’) | Partial | 31 NULL values out of 65 well. These wells are classified as ‘abandoned’ |

The analysis highlights substantial heterogeneity in the representation of well start dates across countries and jurisdictions:

- **True spud dates** are explicitly available, well-defined in only three country datasets (Alaska, Greenland and Norway).
- **Missing spud dates** are frequently linked to specific well statuses indicating that drilling never commenced (e.g. expired or cancelled permits), suggesting that NULL values often carry meaningful information.
- **Proxy temporal attributes**, especially in Canadian datasets, are widely used and often complete, but they may represent administrative or regulatory events rather than the physical start of drilling operations.

**Implications for the interpretation of overlap analyses and potential multitemporal analysis**

This assessment confirms that the oil and gas datasets used in the study include both active and historical wells, including wells for which drilling was authorized but never initiated. ‘NULL’ spud date values are not random but are often linked to well status, providing important contextual information.

While most datasets include both active and historical wells, the timing of drilling initiation cannot be consistently reconstructed across all countries. As a result, given the heterogeneity and incompleteness of temporal information across wells, an Arctic-scale consistent multitemporal analysis of spatial overlaps between oil and gas activities, ecologically sensitive areas, and Indigenous Peoples’ lands is not feasible.

Specifically:

- The absence of harmonized spud dates prevents reliable reconstruction of when drilling occurred in relation to ecological or Indigenous spatial layers.
- The mixed use of true spud dates and administrative proxies introduces temporal uncertainty that cannot be resolved uniformly.

In light of these constraints, we decide to adopt a spatially explicit but temporally inclusive approach, by using the complete datasets published by third parties. Spatial intersections are performed using the geographic information of wells regardless of their current operational status or precise timing, capturing both historical and current exposure of ecologically sensitive areas and Indigenous lands to oil and gas development. For example, we decided to include plugged and abandoned wells that are no longer active. Properly plugged wells are generally considered safer than wells classified as abandoned without explicit plugging information, although they may still have residual environmental impacts due to legacy infrastructure or long-term integrity risks and are therefore relevant for spatial analysis.

While studies on inactive and abandoned wells report the potential for hydrocarbon leakage over time, persistent soil and habitat disturbance due to remaining infrastructure and ground modification, residual emissions such as methane if plugs degrade or fail, and land-use constraints or monitoring requirements associated with abandoned [1, 2, 3], well sites these often refer to wells without documented plugging; therefore, plugged wells are expected to pose substantially lower risk. Therefore, the inclusion of both plugged and abandoned wells and abandoned wells without documented plugging in spatial analyses is appropriate for addressing potential cumulative and legacy impacts of both active and inactive (abandoned) wells, but it does not attempt to quantify temporal dynamics such as the temporal relationship between the establishment of PAs and the activity of oil and gas wells.

**References:**

1. Williams JP, Regehr A, Kang M. Methane Emissions from Abandoned Oil and Gas Wells in Canada and the United States. Environ Sci Technol. 2021 Jan 5;55(1):563–70.
2. Kang M, Boutot J, McVay RC, Roberts KA, Jasechko S, Perrone D, et al. Environmental risks and opportunities of orphaned oil and gas wells in the United States. Environ Res Lett. 2023 June;18(7):074012.
3. Alsubaih A, Sepehrnoori K, Delshad M, Alsubaih A, Sepehrnoori K, Delshad M. Environmental Impacts of Orphaned and Abandoned Wells: Methane Emissions, and Implications for Carbon Storage. Applied Sciences [Internet]. 2024 Dec 11 [cited 2025 Dec 24];14(24). Available from: <https://www.mdpi.com/2076-3417/14/24/11518>

## Identification and temporal interpretation of “areas under bid”

### Motivation

This analysis clarifies the identification and interpretation of this study’s “areas under bid”. Specifically, it clarifies how areas under bid were defined, whether bidding represents a permanent or time-bound process, and how datasets published by third parties were interpreted. Because bidding rounds are often periodic and not continuous, a clear definition is required to assess the validity of spatial intersections with ecologically sensitive areas and Indigenous Peoples’ lands. Therefore, we reviewed the sources, temporal scope, and regulatory meaning of bidding-related spatial datasets used in the study for each country.

In the context of oil and gas governance, *areas under bid* refer to geographic areas that are made available by governments or regulatory authorities for competitive bidding by companies seeking exploration or production rights. In simple terms, these areas may be offered through different mechanisms [1]:

- Permanent or open-door bidding systems, where areas are continuously available unless withdrawn (e.g. due to licensing or regulatory restrictions);
- Periodic bidding rounds, typically annual or multi-annual, where areas are offered only during specific time windows;

Importantly, the presence of an area in a bidding dataset does not imply active operations, nor does it necessarily imply current interest by companies (e.g., historical bidding rounds, representing areas offered in the past but no longer active). All these details are addressed in the geographical definition of areas under bid included in S8 Table.

### Assessment criteria

Each national dataset was evaluated according to the following criteria:

1. **Bidding mechanism or status**
   - Permanent/open-door offering
   - Periodic (e.g. annual) bidding rounds
   - Historical (no longer active)
2. **Temporal validity** → Whether the dataset represents:
   - currently available bidding areas,
   - a snapshot of a specific bidding round,
   - legacy information from past rounds.
3. **Data availability and continuity** → Whether bidding data are still publicly available and regularly updated.
4. **Interpretation for spatial analysis** → Whether areas under bid should be interpreted as:
   - persistent exposure to potential future development,
   - historical exposure only.

### Results: national and regional interpretations of bidding areas

**Table B – Interpretation of “areas under bid” datasets by country/region**

| **Country / Region** | **Bidding mechanism** | **Temporal nature of CAFF data** | **Data availability** | **Interpretation and notes** |
| --- | --- | --- | --- | --- |
| Alaska (USA) | Continuously available for leasing unless restricted | Persistent | Available | Areas continuously available for leasing unless restricted. Dataset represents ongoing potential for future bidding |
| Greenland | Periodic bidding rounds (historical) | No longer active | Not available | Oil and gas bidding discontinued following policy decisions. No active bidding areas and historical bidding data no longer available in accessible geospatial datasets. |
| Norway | Mixed system: permanent and annual rounds | Dynamic | Available | Predefined areas available for annual APA with spatial extent updated over time as blocks are awarded or reclassified; excludes licensed production blocks. |
| Russia | – | – | No data | No publicly available spatial data on bidding areas |
| Canada (general) | Periodic bidding rounds, province-specific | Various | Available | Highly heterogeneous; varies by province and over time. Datasets represent past bidding rounds captured as static layers; no longer active. |
| Canada – Newfoundland & Labrador | Periodic bidding rounds | Legacy | Available | Mostly outside of the CAFF region, CAFF data represents past offshore bidding rounds. Active rounds frequent outside the CAFF boundaries. |
| Canada – Yukon | Periodic bidding rounds | Legacy | Available | Legacy bidding rounds only |
| Canada – Northern Canada | Periodic bidding rounds | Historical | Available | Dataset represents areas from past bidding rounds only; no current or active bidding areas exist due to federal moratoria, and the data do not reflect present-day leasing availability. |

This review highlights substantial heterogeneity in how bidding areas are defined and maintained across jurisdictions:

- Some regions (e.g., Alaska) operate areawide leasing systems, where areas identified for leasing represent a persistent regulatory availability for potential future development rather than active or time-bound bidding events.
- Other regions (e.g. Norway) combine permanent offerings with periodic bidding rounds, resulting in a spatially and temporally dynamic bidding landscape.
- Several jurisdictions (e.g., Greenland and Northern Canada) include historical bidding areas that reflect past policy frameworks rather than current leasing opportunities. These datasets are retained in the analysis because they delineate areas that have been previously identified, based on geological and regulatory considerations, as having potential for hydrocarbon exploration, even though no active bidding is currently taking place.
- In some cases (e.g. Russia), spatial bidding data are not publicly available.

**Implications for the interpretation of overlap analyses and potential multitemporal analysis**

Spatial intersections between areas under bid, ecologically sensitive areas, and Indigenous Peoples’ lands were conducted to identify areas where oil and gas development has been considered in the past through formal regulatory processes, regardless of whether bids are current or historical.

The analysis does not assume continuous or current bidding activity in all jurisdictions. Instead, areas under bid are interpreted as indicators of regulatory openness to oil and gas development at some point in time, reflecting locations that have been delineated based on geological and policy considerations. As such, these areas are used as a proxy for spatial patterns of potential future interest in oil and gas development, albeit with a lower level of development certainty and risk than granted concessions or licenses, consistent with the geographical definitions presented in S8 Table.

**References**

1. Tordo S, Johnston D, Johnston D. Petroleum exploration and production rights: allocation strategies and design issues. World Bank Working Paper No. 179 [Internet]. Washington (DC): World Bank; 2010 [cited 2025 Dec 23]. Available from: <http://documents.worldbank.org/curated/en/785881468336848695>

## Temporal attributes and licence types in oil and gas concession datasets

### Motivation

In this analysis, we assessed the availability of concession datasets across countries and we further clarified the interpretability of the methods and the temporal validity of spatial overlap analyses involving concessions. Specifically, we examined whether these datasets contain (i) explicit licence start dates and end dates, (ii) information on activity status and (iii) information on the type of licence (e.g. exploration or production), which are critical for interpreting the nature of oil and gas activities and their spatial relationships with other priorities. Specifically, this assessment aims to clarify whether concession datasets provide sufficient temporal resolution to support time-explicit overlap analyses with ecologically sensitive areas and Indigenous Peoples’ lands.

### Assessment criteria

For each national or regional concession/licence dataset, we evaluated:

1. **Presence of temporal attributes.** Whether licence start dates (and, where available, expiry dates) are explicitly provided.
2. **Activity status available.** Whether licenses can be classified as ‘active’ or ‘inactive’ or ‘other status’
3. **Licence type information.** Whether licences can be classified as exploration, production, or other categories (e.g. significant discovery, pioneer lease).
4. **Temporal interpretability.** Whether the available temporal attributes allow licences to be reliably positioned in time in a way that is comparable across jurisdictions.

### Results: temporal coverage and licence types by country/region

#### Table C – Availability of temporal attributes and licence types in concession/licence datasets used in this study.

| **Country / Region** | **Authorization / grant date available** | **Activity status available** | **Licence type information** | **Interpretation and limitations** |
| --- | --- | --- | --- | --- |
| **Alaska – ‘Alaska Division of Oil and Gas’ data** | Yes (Attribute ‘Effective Date’) | Yes (Attribute CaseStatus) | Yes (Attribute CaseType) | Temporal information available; licence status and type documented |
| **Alaska – ‘NPRA’ data** | Yes (Attribute ‘YEAR’) | Not specified | Not specified | Temporal information available; licence type/status unclear |
| **Canada – British Columbia** | No explicit licence start date | Not specified | Production only | Dataset represents production fields; no temporal analysis possible |
| **Canada – Yukon** | Yes (Attribute ‘ISSUED’, ‘TERM’ and ‘EXPIRY_DAT’) | Yes (‘Attribute’ STATUS: active, expired, surrender, inactive) | Yes (Attribute ‘TYPE’: exploration licence, permit, lease, significant discovery licence) | Temporal information available; licence status and type documented |
| **Canada – Alberta** | Yes (Attribute ‘DATE’) | Yes | Yes | Temporal attribute available; licence status and type documented at:  <https://static.aer.ca/prd/data/layouts/AER-Order-System-BOS-DataTXT-Layout_2025.pdf>  And <https://maps.aer.ca/osm/index.html> |
| **Canada – Northwest Territories (Crown–Indigenous Relations dataset)** | Yes (Attribute ISSUE_DTE, EXPIRY_DTE) | Yes. Implicit (via expiry date).  Offshore areas under moratoria: <https://www.rcaanc-cirnac.gc.ca/eng/1535571547022/1538586415269> and <https://www.pm.gc.ca/en/news/statements/2016/12/20/united-states-canada-joint-arctic-leaders-statement> | Yes (Attribute ‘AGREETYP’: Attribute offshore exploration and significant discovery licences) | Temporal and licence-type information available |
| **Canada – Northwest ‘Territories Mineral and Petroleum Resources Division’ data** | Yes (Attribute ISSUE_DTE, EXPIRY_DTE) | Yes. Implicit (via expiry date).  Offshore areas under moratoria: <https://www.rcaanc-cirnac.gc.ca/eng/1535571547022/1538586415269> and <https://www.pm.gc.ca/en/news/statements/2016/12/20/united-states-canada-joint-arctic-leaders-statement> | Yes (Attribute ‘AGREETYP’: commercial discovery declaration, pioneer lease, production licence, significant discovery declaration, significant discovery licence) | Temporal and licence-type information available |
| **Canada – Nunavut** | Yes (Attribute ISSUE_DTE, EXPIRY_DTE) | Yes. Implicit (via expiry date)  Offshore areas under moratoria: <https://www.rcaanc-cirnac.gc.ca/eng/1535571547022/1538586415269> and <https://www.pm.gc.ca/en/news/statements/2016/12/20/united-states-canada-joint-arctic-leaders-statement> | Yes (Attribute ‘AGREETYP’: commercial discovery declaration, pioneer lease, production licence, significant discovery declaration, significant discovery licence) | Temporal and licence-type information available |
| **Canada – Newfoundland & Labrador** | Yes (Attribute ‘Effective’) | Yes. Active | ‘Exploration licenses’, ‘Production licenses’, ‘Significant discovery licences’ divided in different datasets | Only significant discovery licences intersect CAFF boundaries |
| **Norway** | Yes (Attribute ‘dtGranted’) | Yes (Attribute ‘STATUS’) | Production licences only | Temporal information available; licence status and type documented |
| **Greenland** | Yes (Attribute Start date, Expiry date) | Yes. Implicit (via expiry date) | Exploration licences only | Temporal information available; licence status and type documented |
| **Russia** | No | No | No | No publicly available concession or licence data. Catalogued as ‘fields’ (areas above a hydrocarbon accumulation) by GOGI |

**Implications for the interpretation of overlap analyses and potential multitemporal analysis**

This assessment shows that, unlike wells and bidding areas, concession and licence datasets more frequently include explicit start dates and licence type information. However, several limitations remain:

- Temporal attributes are not harmonized across countries (e.g. Effective Date, ISSUED, dtGranted), and therefore their regulatory meaning may vary.
- Licence datasets represent different activity phases across jurisdictions (exploration-only, production-only, or mixed), limiting cross-country comparability.
- Key countries (e.g. Russia) lack institutional concession data.

The review of concession datasets demonstrates that, although temporal and categorical information is available for several regions and while regional or country-specific multitemporal analyses may be feasible, the heterogeneity in data structure, licence types, and temporal semantics precludes a fully harmonized, arctic-scale multitemporal overlap analysis across countries.

When considered jointly with well-level and bidding datasets, the concession analysis confirms that:

- Temporal information is unevenly distributed across data types and jurisdictions;
- No single dataset provides complete, harmonized temporal coverage of oil and gas activities at the global scale.

**Therefore, the spatial overlaps presented in this study do not necessarily imply a current oil and gas activity in ecologically sensitive areas or Indigenous Peoples’ lands, but rather reflect cumulative and regulatory exposure over time.**

**S6 Table. Definitions and descriptions for ‘concessions’ and ‘areas under bid’**

| **Term** | **Legal definition** | **Geographical definition used in this study** |
| --- | --- | --- |
| Concessions | A concession is a legal arrangement through which a state grants an oil and gas company (or consortium) the exclusive right to explore for and produce hydrocarbons within a defined geographic area and for a specified period of time, according to specific technical, economic and environmental criteria.. Under a concession regime, the investor assumes all exploration, development, and production risks and costs, typically pays license fees or bonuses, and compensates the state through royalties and taxes on production. Ownership of petroleum in situ remains with the state until hydrocarbons are produced, at which point title transfers to the investor at the wellhead [1]. | In this study, we use the term 'concession' to refer to spatial units delimiting areas designated for oil and gas exploration and production activities across the Arctic countries. These categories (such as fields, leases, and licenses) vary considerably in their legal meaning and administrative function across national and sub-national contexts (see S1 Appendix). Therefore, to ensure comparability across jurisdictions, we adopt a geographical rather than legal definition, treating these heterogeneous units as spatial representations of areas where extractive activities are occurring or may occur in the near future.  Although exploration and production areas come from different legal categories, we treat them together as a single geographical dataset for the purpose of spatial analyses. This decision reflects the fact that it is not always possible to clearly distinguish between exploration and production licenses. Accordingly, the ‘concessions’ used in this study represent areas where extractive activity occurred, is occurring or could occur in the future. As such, they reflect geographies of potential socio-environmental perturbations and indicate where spatial overlaps may arise (or may already exist) between extractive interest, conservation priorities, and Indigenous Peoples’ rights, as already conceptualized in previous studies [2, 3, 4]  While exploration and production areas are integrated into a single dataset for spatial analyses, the distinction between these two stages is explicitly considered and discussed in the interpretation of results. The resulting dataset is not exhaustive; however, it provides a reasonable representation of the spatial distribution of major oil and gas industry activities and interests in the Arctic. Importantly, the data used in this study to represent concessions are consistent with those employed in previous studies and data-collection efforts, including OGIM [5] and GOGI [6] ensuring methodological continuity and comparability with existing literature. |
| Areas under bid | In the hydrocarbons sector, a bidding area is a geographically defined onshore or offshore area, delineated by the competent public authority and offered to investors through a competitive allocation process, within which exploration and/or production rights for oil and gas resources are awarded to the winning bidder according to predefined technical and fiscal criteria [1, 7] | In this study, ‘areas under bid’ are spatial units that were officially offered or kept open for oil & gas licensing by national authorities at the time of data collection, regardless of the specific licensing mechanism (e.g., annual rounds, permanent offer regimes, or historically active bidding areas) [1]. Due to the heterogeneity of national regulatory systems and the uneven availability and comparability of official data, this category captures all areas in which governments signaled potential future extractive opportunities by making them available for bidding, rather than their specific legal status. Therefore, as with the concessions, the category is defined in geographical rather than legal terms.  This conceptualization is grounded in the assumption that areas offered for bidding but not awarded do not lose their extractive potential: they remain under state control and may be re-offered in subsequent licensing rounds or reconfigured into new blocks. In this sense, areas under bid provide a spatial indication of where oil and gas activities are more likely to develop in the future, albeit with a lower level of development certainty and risk compared to concessions (as geographically defined).  Moreover, this conceptualization is consistent with previous work by Harfoot and colleagues, who defined areas under bid as “future exploration blocks” (i.e locations “where tenders were invited as of” a specific time, or “locations where future exploration and possible hydrocarbon production is likely to take place. Future-exploration blocks thus represent the more distant future of oil and gas exploration and production impacts, compared with licensed blocks.”) [4] |

**References**

1. Tordo S, Johnston D, Johnston D. Petroleum exploration and production rights: allocation strategies and design issues. World Bank Working Paper No. 179 [Internet]. Washington (DC): World Bank; 2010 [cited 2025 Dec 23]. Available from: <http://documents.worldbank.org/curated/en/785881468336848695>
2. Cuba N, Bebbington A, Rogan J, Millones M. Extractive industries, livelihoods and natural resource competition: Mapping overlapping claims in Peru and Ghana. Appl Geogr. 2014 Oct 1;54:250–61.
3. Codato D, Pappalardo SE, Diantini A, Ferrarese F, Gianoli F, De Marchi M. Oil production, biodiversity conservation and indigenous territories: Towards geographical criteria for unburnable carbon areas in the Amazon rainforest. Appl Geogr. 2019 Jan 1;102: 28–38.
4. Harfoot MBJ, Tittensor DP, Knight S, Arnell AP, Blyth S, Brooks S, et al. Present and future biodiversity risks from fossil fuel exploitation. Conserv Lett. 2018;11(4):e12448.
5. O’Brien M, Omara M, Himmelberger A, Gautam R. Oil and Gas Infrastructure Mapping (OGIM) database dataset on the Internet. Zenodo; 2025. Available from: https://zenodo.org/records/15103476
6. Rose K, Bauer J, Baker V, Bean A, DiGiulio J, Jones K, Justman D, Miller RM, Romeo L, Sabbatino M, Tong A. Development of an open global oil and gas infrastructure inventory and geodatabase. NETL Technical Report Series; NETL-TRS-6-2018. Albany (OR): U.S. Department of Energy, National Energy Technology Laboratory; 2018. 594 p. doi:10.18141/1427573
7. Cameron PD, Stanley MC. Oil, Gas, and Mining: A Sourcebook for Understanding the Extractive Industries [Internet]. Washington (DC): World Bank; 2017[cited 2025 Dec 23]. doi: 10.1596/978-0-8213-9658-2.
